# An *in silico* framework for dissecting the mechanistic origins of *in vivo* recorded neuronal activity

**DOI:** 10.1371/journal.pcbi.1014617

**Published:** 2026-08-21

**Authors:** Bjorge Meulemeester, Arco Bast, María Royo, Rieke Fruengel, Su Saka, Foivos Kastrinakis, Marcel Oberlaender

**Affiliations:** 1 Max Planck Institute for Neurobiology of Behavior - caesar, Bonn, Germany; 2 International Max Planck Research School (IMPRS) for Brain and Behavior, Bonn, Germany; 3 Department of Integrative Neurophysiology, Center for Neurogenomics and Cognitive Research, Vrije Universiteit Amsterdam, Amsterdam, the Netherlands; Research Centre Jülich: Forschungszentrum Jülich GmbH, GERMANY

## Abstract

How can we identify the mechanistic origins of the electrophysiological activity that is recorded from neurons in the living brain? A promising strategy for addressing this question is to generate biologically realistic models of *in vivo* recorded neurons, and simulate how they transform synaptic inputs from the network into their observed neuronal activity. For this purpose, we here provide our approaches for the generation, simulation, and analysis of network-embedded neuron models as an open source, fully documented and freely available software environment: In Silico Framework (ISF). ISF is centered around the concept of achieving “model consensus” about the mechanistic origins of *in vivo* recorded activity across biologically diverse sets of models. To achieve such model consensus, ISF offers three key workflows. First, ISF enables users to generate models that are equally well constrained by empirical data at subcellular, cellular and network scales, while the set of models as a whole is constructed to exhibit maximally diverse parameters, spanning the full ranges permitted by the empirically observed biological variability at each scale. Second, ISF enables users to identify those subsets of model configurations that predict the *in vivo* observations without being tuned to do so. Third, for each of those model configurations, ISF enables users to identify which mechanisms at subcellular, cellular and network scales are necessary to predict the *in vivo* observations, and which mechanisms are dispensable. Thereby, ISF can reveal which mechanisms are common across model configurations, and whether the diversity of model configurations could account for the variability of the *in vivo* observed activity across animals, cells and trials. In essence, by achieving such model consensus, ISF predicts mechanisms that are robust across biological variability, and which may hence indeed be used *in vivo*. Finally, ISF enables users to derive model consensus for *in silico* manipulations, to identify which experimental strategies would be best suited to test the predicted mechanisms *in vivo*. We exemplify how we have used this iterative *in silico* - *in vivo* approach of ISF to dissect the mechanistic origins of sensory responses in the barrel cortex. By making ISF available as a standalone online resource, we believe it will facilitate the generation, simulation and analysis of models that reveal mechanistic origins of *in vivo* recorded activity beyond the barrel cortex for which it was originally designed.

## Author summary

Using computational modelling to disentangle the mechanisms that underlie neuronal activity *in vivo* remains challenging. Here we present In Silico Framework (ISF) – an open source, fully documented and freely available software environment that enables the generation, simulation, and analysis of sets of biologically detailed neuron-network models. ISF constrains each model with empirical data at the subcellular, cellular, and network scales. Crucially, however, the set of models as a whole is constructed to exhibit maximally diverse parameters, spanning the full ranges permitted by empirically observed biological variability. As we showed previously for neurons in the rat somatosensory (barrel) cortex, ISF can reveal which mechanisms account for *in vivo* observed neuronal activity, which cellular and circuit properties account for the observed variability of this activity, and which *in vivo* experiments could be done to test these in silico predictions. Therefore, we make the source code of ISF available, in combination with a detailed documentation and explanation of all workflows, including interactive tutorials and visualizations from our published work in the barrel cortex.

## Introduction

The electrophysiological activity of each neuron in the brain is the result of a complex interplay between multiple mechanisms at the subcellular, cellular, and network scales. The current landscape of *in vivo* and *in vitro* experimental work provides unprecedented insights into these mechanisms, using techniques for measuring synapse dynamics [1] and locations [2–4], the electrophysiological responses that these synaptic input patterns evoke in single dendrites [5], biophysical and electrophysiological properties of active dendrites [6], morphology reconstructions [7,8], network connectivity [9,10] and activity patterns [11]. It is however highly challenging to investigate the joint, simultaneous and interdependent operation of these intricate neuron and network mechanisms. The generation of bottom-up computational models that integrate these *in vitro* and *in vivo* experimental data on individual mechanisms, could provide an opportunity to disentangle and explain *in silico* how these mechanisms could underlie *in vivo* recorded neuronal activity, such as the generation of action potentials (APs) in response to a sensory stimulus. Ideally, such simulation-based explanations can be verified empirically. These computational models should therefore allow us to generate concrete predictions that can be tested experimentally [12]. Conversely, the new insights from these empirical tests can then be used to further constrain and improve computational models, closing the loop from *in silico*-inspired *in vivo* experiments, to an iterative *in silico*-*in vivo* approach.

While bottom-up models could provide the means for understanding the anatomical and physiological substrates of neuronal activity *in vivo* [13,14], it remains a challenge to build such models [15,16], for at least the following reasons. First, the amount and quality of empirical data required is extensive. These bottom-up models need to integrate data on each mechanism that is hypothesized to be relevant for a particular *in vivo* observation. To explain *in vivo* activity, this includes at least the neuron’s morphology, intrinsic physiology, biophysical composition, connectivity to presynaptic neurons — ideally at the dense electron-microscopic (EM) level, and *in vivo* neuronal activity during the experimental paradigm to be explained. Acquiring these data for building a bottom-up model is no small feat, as it requires to perform many experiments with different techniques that measure properties of the same (type of) neurons from subcellular (e.g., biophysics) to network scales (e.g., connectivity). Second, it is not obvious how to integrate such independently acquired data into a coherent model. Measurements at each scale generally exhibit large variability between animals, and across neurons of the same type, and even across trials of the same experiment. Thus, independent of the quality and quantity of individual data points, it is not obvious how to puzzle these heterogeneous data together such that the resulting model provides an accurate representation of the neuronal and network properties *in vivo*. Third, the same activity can be the result of different implementations. It has been shown that even in simple neural systems, there may be different ways to implement a given *in vivo* function [17]. Even a perfectly data-constrained model will still be a single model, and a single model represents only one out of the possible and diverse ways an *in vivo* function could be implemented. Due to this parameter degeneracy, a single model can not be used to confidently quantify the relevance of any individual mechanism for neuronal output, and it would therefore be of limited use to make empirically testable predictions. In sum, for computational models to provide experimentally testable predictions on how diverse biological mechanisms could underlie an *in vivo* observation, they should be built bottom-up from high-quality data, and span a wide range of possible parameter configurations so that they capture biological diversity and parameter degeneracy. This categorically shifts the focus away from optimizing a model so that it reproduces an *in vivo* observation, and towards the generation of multiple, diverse, but equally well-constrained models that reproduce the *in vivo* observation without being tuned to do so. These sets of models capture not just one way, but many different ways in which a particular *in vivo* observation could in principle be implemented.

Over the past two decades, we have aimed to construct precisely such bottom-up models for the rat barrel cortex that address the challenges mentioned above. These models account for the neuron’s empirically observed morphology; intrinsic somatic, dendritic, and axonal physiology; dense synaptic connectivity; and synaptic physiology. We activated these multi-scale models using *in vivo* recordings from identified presynaptic cells in thalamus and barrel cortex under the same experimental conditions that we sought to explain. We generated many equally realistic models that span the empirically observed ranges of parameters at each scale. When activating these models to mimic an *in vivo* experiment, there are no free parameters to be optimized. Instead, each model configuration that predicts activity consistent with the *in vivo* observation is considered a valid model. We then developed strategies to test which mechanisms these diverse models employ to produce the observed *in vivo* activity. When such models converged on common mechanisms underlying the observed activity—i.e., reached consensus about its origins—across a wide diversity of parameters, then these mechanisms were likely to be predictive of those used *in vivo*.

**Fig 1:**
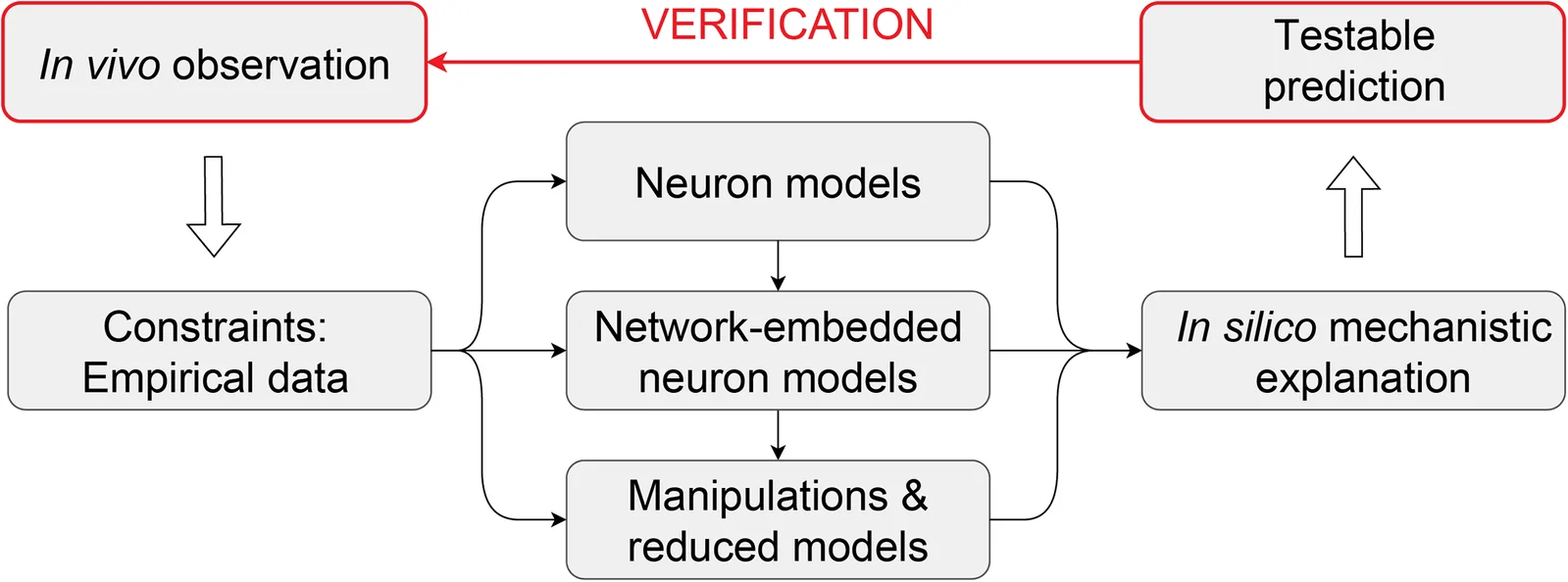
Organigram of ISF. To explain *in vivo* observed activity, empirical data is used to build and constrain neuron models and network-embedded neuron models. The *in vivo* observed activity itself is not used to tune these models. Model reduction and *in silico* manipulations are used to predict which mechanisms could underlie *in vivo* activity. From these *in silico* predictions, experimentally testable hypotheses can be derived that can be verified *in vivo*.

To test whether these predicted mechanisms are indeed used *in vivo*, we explored the effects of experimentally achievable (e.g., pharmacological) *in silico* manipulations that specifically target the predicted mechanisms. If an *in silico* manipulation produced a similar effect across the diverse set of model configurations, and this effect was strong enough to be measurable *in vivo*, it constituted a suitable experimental strategy to test the model prediction. We consequently carried out this experiment *in vivo*. Our approach thus constitutes an iterative *in silico*–*in vivo* procedure (Fig 1).

We demonstrated previously [18–21] that our bottom-up modeling approach can indeed achieve model consensus, and hence provide insight into the mechanistic origins of *in vivo* recorded neural activity in the rat barrel cortex. For this reason, we present our In Silico Framework (ISF) as a freely available, open-source and well-documented [22] codebase for the generation, simulation, and analysis of diverse and biologically detailed network-embedded neuron models. ISF has an explicit focus on generating a diverse set of models, all of which can account for an *in vivo* observation, and all of which are equally well constrained by empirical data at subcellular, cellular and network scales. We provide detailed explanations of all workflows, and how to install and run ISF on different infrastructures and operating systems. Moreover we provide detailed tutorials and visualizations [23] from our previous work on explaining sensory-evoked responses in the barrel cortex [19–21,24,25], which will help to adapt our approaches to different systems and to extend it to different experimental paradigms and questions in the barrel cortex.

## Results

The purpose of ISF, as outlined by the following three sections, is to mechanistically explain the electrophysiological activity of individual neurons during a specific *in vivo* experiment. To do so, ISF generates, simulates, and analyzes sets of biologically detailed network-embedded neuron models that are well-constrained by empirical data at the subcellular, cellular and network scales. Simultaneously, the set as a whole is constructed to exhibit diverse parameters spanning the ranges permitted by empirically observed biological variability across animals, neurons of the same type, and trials of the same experiment.

### Generating diverse sets of neuron models that capture intrinsic physiology

**Fig 2:**
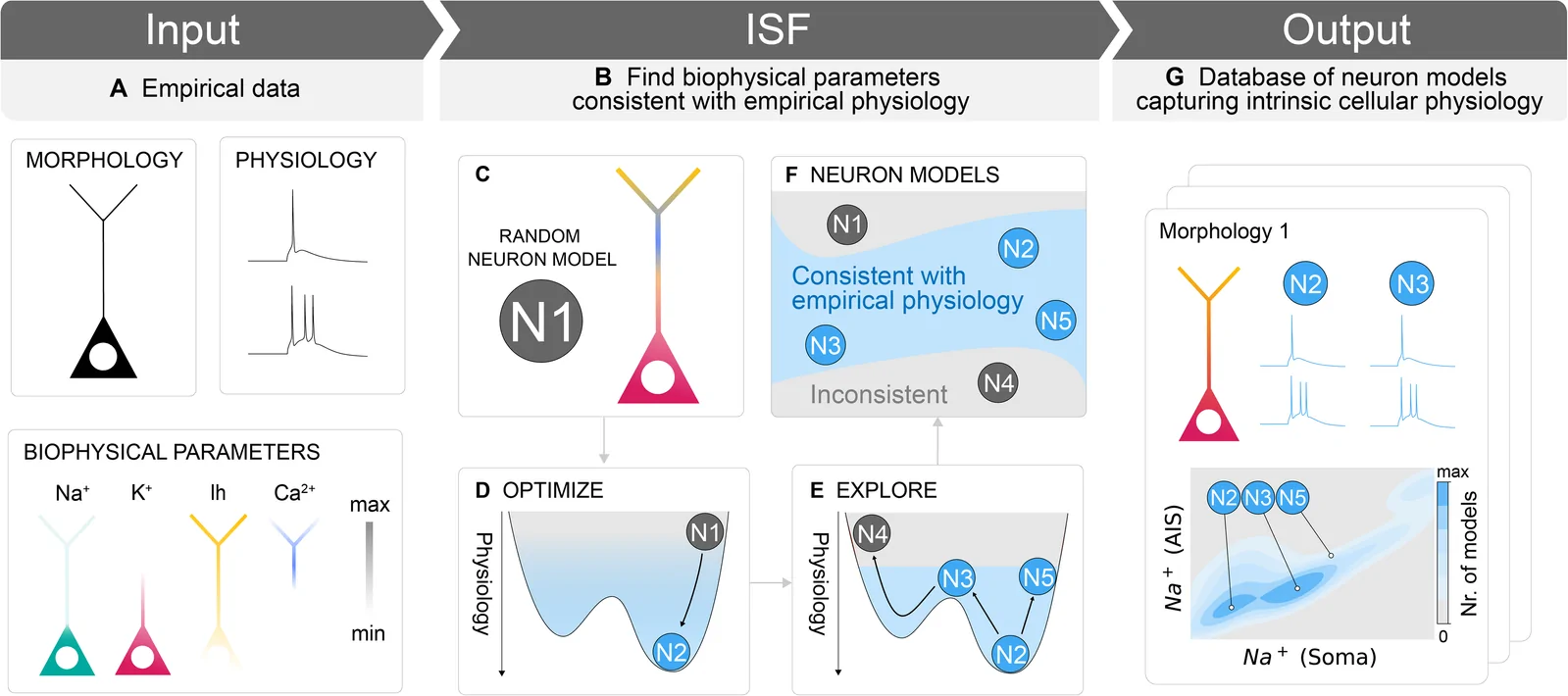
Generating diverse sets of biologically detailed neuron models that capture intrinsic cellular physiology. (A) A morphology and empirical measurements on the neuron’s response to current injection stimuli and biophysical parameters are required input data. (B) ISF generates neuron models from the input data. (C) ISF takes a random neuron model N1 whose parameters lie within empirical bounds. (D) ISF optimizes the biophysical parameters of N1 until they can reproduce the empirically observed response to the same input stimuli, creating a new neuron model N2. (E) ISF uses neuron model N2 as seed point to explore the space of biophysical parameters and find diverse neuron models (e.g., N3 and N5). (F) ISF finds models that are consistent with the empirical physiology, while being biophysically diverse. (G) The output of this workflow is a large database of empirically constrained, yet biologically diverse neuron models for each morphology.

The purpose of this workflow is to generate neuron models that capture the intrinsic physiology of their cell type. The empirical data this workflow requires as an input are (1) a morphology reconstruction, (2) electrophysiological recordings that capture the cell type’s intrinsic physiology, and (3) bounds on the biophysical parameters that describe the neuron’s passive and active properties (Fig 2A). All parameters listed in ISF’s neuron parameter file [26], including spatial distributions of ion channels and their kinetics, can be used for this workflow (see Methods for more details).

To generate neuron models from this input (Fig 2B), ISF converts the morphology into a multi-compartmental model, inserts ionic mechanisms such as ion channels and a calcium buffer, (Fig 2C), performs *in silico* current injection experiments, and optimizes the biophysical parameters until the *in silico* response lies within the empirically observed response distribution (Fig 2D). This produces a single neuron model that is constrained by empirical data in terms of morphology, biophysical parameters, and intrinsic physiology. This single neuron model is then used as a seed point for exploring the biophysically diverse parameter space (Fig 2E) by which the model produces responses consistent with the empirical observations for each current injection stimulus (Fig 2F). This procedure is then repeated for a set of neurons that capture the morphological diversity of the cell type.

As an output, ISF produces a database of morpho-electrically diverse neuron models whose biophysical parameters lie within the empirically observed ranges, and whose intrinsic electrophysiology is consistent with empirical observations (Fig 2G).

### Generating diverse sets of network-embedded neuron models that capture *in vivo* conditions

The purpose of this workflow is to generate synapse locations and activation patterns onto neuron models that mimic the specific conditions of an *in vivo* experiment. For clarity, we refer to the neuron models as postsynaptic, while neurons providing input to neuron models are presynaptic. ISF generates synaptic input to neuron models in two stages: (1) it embeds a neuron model into a network model, and (2) assigns *in vivo* like activity patterns to each synapse.

First, the purpose of embedding a neuron model in a network model is to provide constraints on the number of synapses, the location of these synapses onto the postsynaptic neuron (i.e., including the soma, dendrites, and axon initial segment), and on their neurons of origin in the network. Our strategy is to generate a model of the dense neuropil structure, place the neuron model into the neuropil model, and provide wiring rules on how neurons in the dense neuropil model form synapses with the type of the model neuron. To capture variability in network connectivity, one could change the embedding location of the neuron model, the wiring rules, or generate multiple samples per location if the wiring rules have stochasticity. As a result, ISF provides a set of network-embedded neuron models that are consistent with empirical connectivity data, but which differ in the number and placement of synapses, as well as the neurons from which these synapses originate. This set of network-embedded neuron models provides a way to test whether and how variability in connectivity impacts the simulation results for *in vivo*-like input patterns.

We reported previously how to generate neuropil models and wiring rules that produce connectivity patterns that are consistent with those observed empirically in dense EM data [10,24,27], or which are derived directly from dense EM data [24,28]. Moreover, we provide examples on how to embed neuron models into a dense neuropil model of the barrel cortex [29,30]. This embedding procedure is set as the default option in ISF. Alternatively, users can explicitly define connectivity themselves by generating the respective connectivity files [31,32].

**Fig 3:**
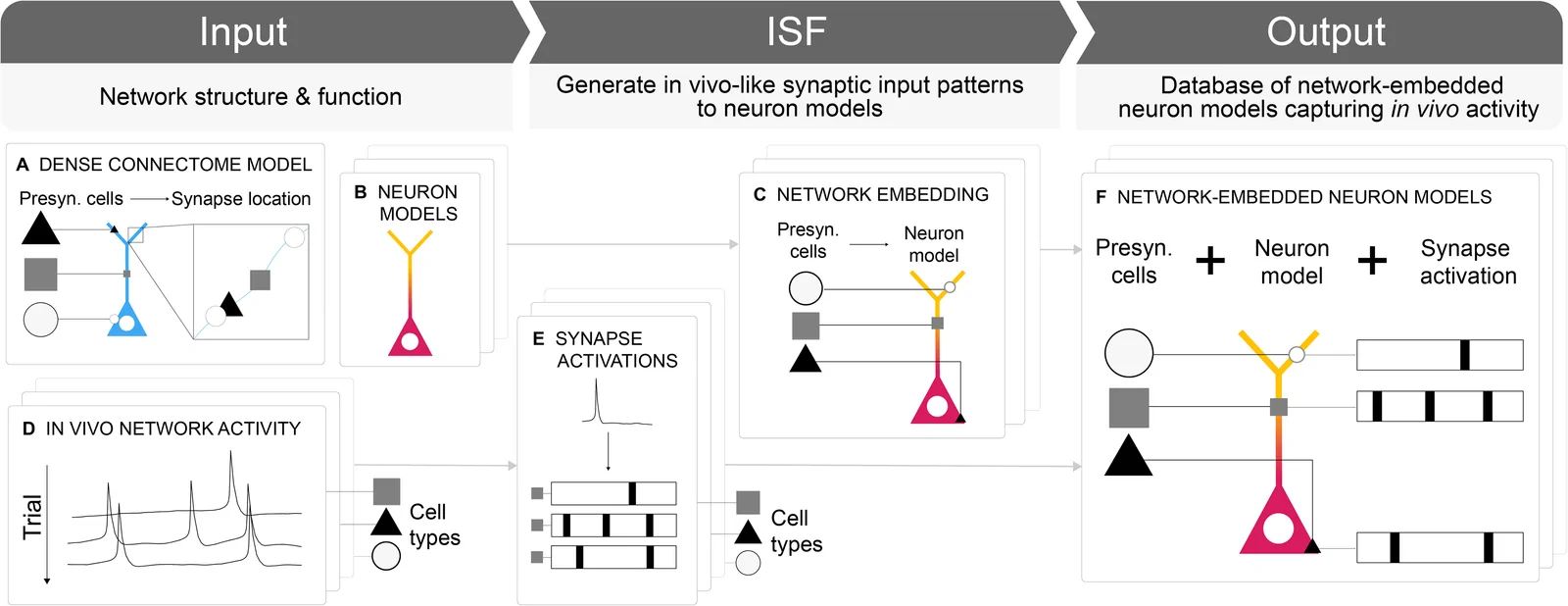
Generating diverse sets of biologically detailed network-embedded neuron models that capture *in vivo* activity. Required empirical input data consists of (A) a dense connectome model, (B) neuron models, and (D) activity recordings of cell from all populations during an *in vivo* stimulus. (C) ISF creates multiple network embeddings per neuron model, and (E) generates multiple synapse activation patterns based on the empirically observed network activity per network-embedded neuron model. (F) The output is a database of empirically constrained, yet biologically diverse network-embedded neuron models.

Second, to constrain the activation patterns of these synapses, ISF requires empirical data on synaptic strength in the form of unitary post-synaptic potential (uPSP) measurements, and activity rates for each cell type under the *in vivo* condition being investigated (Fig 3D). ISF then scales synaptic weights such that the resulting cell type-specific uPSP distributions match those observed empirically, and activates presynaptic neurons according to the empirically observed cell type-specific activity rates during the *in vivo* condition of interest (Fig 3E). ISF thereby generates spatiotemporal synapse activity patterns that are constrained by anatomical and functional data to mimic the synaptic input received *in vivo*. As a result, ISF produces a database of network-embedded neuron models that cover a broad diversity in terms of morphology and biophysics of the neuron, strength and spatial distribution of synapses, and presynaptic origin and timing of synapse activations (Fig 3F).

### Predicting the origins of *in vivo* activity from simulations of diverse network-embedded neuron models

To simulate this diverse set of network-embedded neuron models, ISF numerically integrates the dynamics of synapse activations, ion channels and intracellular *Ca*^2+^-buffering, and the electrical properties of the membrane. These simulated results are then compared to measurements of *in vivo* observed activity, such as ongoing and sensory-evoked firing rates, receptive field shapes, occurrences of bursts of APs, dendritic spikes, or any other feature of *in vivo* activity. Importantly, model parameters are not tuned to achieve this match. Instead, all model parameters were already constrained by empirical data that is independent of the *in vivo* observation in previous steps (see “Input” of Figs 2 and 3). In other words, the empirical data used to constrain these network-embedded neuron models are categorically different from the *in vivo* observation to be explained, and the *in vivo* observation is at no point used as a constraint for parameter adjustments.

Can these models produce the *in vivo* observation under investigation? Each network-embedded neuron model that can do so provides one hypothesis of how the underlying mechanisms could generate the *in vivo* observed activity, and is selected for further analysis. If, on the other hand, not a single model can reproduce the *in vivo* observed activity, then the model generation requires further refinement. Likely, a biological mechanism that is important for the phenomenon is not present in the models, the empirical constraints are insufficient, or the space of possible network-embedded neuron models is insufficiently explored (see Discussion).

Those network-embedded neuron models that are consistent with *in vivo* observed activity allow us to further explore the possible origins of this activity, and to examine whether these models agree about their origins. When the models converge on common mechanisms underlying the *in vivo* observed activity, across a wide diversity of parameters, it indicates that these mechanisms are robust under the modeled conditions and well supported by the data used to constrain the model, even when this data exhibits intrinsic biological variability. In other words, the models have reached a consensus on how an *in vivo* function is implemented, despite their diversity. Such “model consensus” represents a strong prediction that these mechanisms may be used *in vivo*. Mechanisms that are predicted this way are ideally suited for “closing the loop,” i.e., performing an *in vivo* experiment that directly tests the proposed mechanism. Such an experiment can first be performed *in silico* until an experimental strategy has been found that is capable of revealing the underlying mechanism, and is achievable with the available experimental techniques.

**Fig 4:**
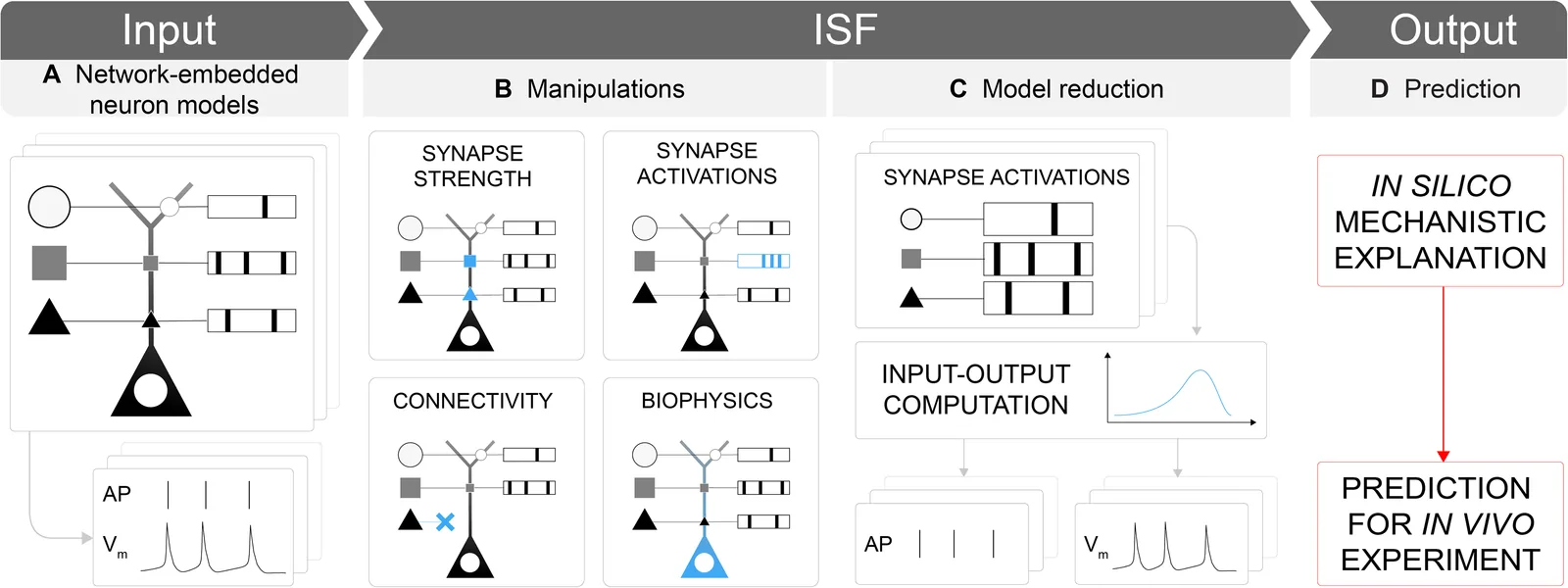
Predicting the mechanistic origins of *in vivo* activity from simulated network-embedded neuron models via model reduction and *in silico* manipulations. (A) A set of biologically diverse network-embedded neuron models and their simulated neuronal responses to sensory-evoked synaptic activity are a required input for further analysis. Both somatic AP times and the somatic voltage trace are shown. (B) Four examples of manipulations to simulated network-embedded neuron models, clockwise from the top left: scaling the strengths of synapses, changing the activation times of specific synapses, changing the biophysical properties of the neuron model, and changing the connectivity between the presynaptic population and the neuron model. Manipulations that target synapse strengths, activations, and connectivity can be manipulated based on their presynaptic cell type, location on the dendrite, at random, individually, or any other property. (C) network-embedded neuron models are reduced to analytically tractable models that capture their input-output computations. (D) Both the manipulations and reduced models aim to provide concrete *in silico* predictions that can be tested experimentally.

For the purpose of investigating the underlying mechanisms and designing empirically testable experiments, ISF provides two additional workflows. The first strategy is to manipulate specific properties of network-embedded neuron models and investigate their effect on their simulated neuronal activity (Fig 4B). The second strategy is to reduce these models to a minimal set of parameters that are sufficient to account for an *in vivo* observation (Fig 4C). Both strategies can be combined as well: it is possible to apply manipulations to reduced models, or apply model reduction to manipulated simulations. The goal of both strategies is to provide empirically testable predictions on how neuron and network mechanisms underlie *in vivo* activity (Fig 4D).

#### Strategy 1: Manipulations.

The purpose of manipulating simulations of network-embedded neuron models is to isolate the relevance of individual mechanisms for the observed *in vivo* neuronal activity. ISF can re-simulate existing simulation runs with the exact same biophysical parameters, synapse locations and synapse activation times, while making any of the following targeted manipulations (see Fig 4B): (1) scaling synaptic weights conditional on their dendritic location, receptor type, presynaptic origin, or individually; (2) changing synaptic activation patterns by the same categories; (3) removing connectivity to presynaptic cells or populations; (4) changing biophysical parameters; (5) any combination of the above.

ISF thereby enables the investigation of the effects of each manipulation across the diverse set of network-embedded neuron models. The effects of these manipulations, and to which degree these manipulations have the same effects despite variations of properties from subcellular to network scales, provides robust predictions for the neuron and network mechanisms, and their interactions, underlying *in vivo* activity. These *in silico* manipulations therefore allow the generation of concrete predictions about underlying mechanisms, and to inform the design of *in vivo* experiments to test these predictions empirically.

#### Strategy 2: Model reduction.

The goal of model reduction is to find an analytically tractable description of the input-output computations of a neuron, with a minimal set of parameters, and to investigate how robust this description is across trials and cells of the same cell type. As we have argued before [20], the specific mathematical form of a reduced model may depend on the *in vivo* condition, neuron model and network model under investigation. For this reason, ISF provides a model reduction package that enables users to derive the mathematical form of a reduced model for their purposes. Specifically, the reduced model package provides three components (see Methods) that allows the user to specify: (1) what features of the input should predict what features in the output, (2) what are the exact mathematical forms of the reduced models, and (3) how to quantify how well the resulting reduced models predict the input-output computations compared to the full mechanistically detailed network-embedded neuron models. ISF additionally provides predefined mathematical forms, evaluation functions, and optimization strategies, e.g., those specified in our example application [20].

The input to this workflow is simulation data generated by a biologically detailed network-embedded neuron model, containing the activation times and locations of the synapses, and the postsynaptic neuron’s response to this input. ISF then extracts the relevant features from the input and output, as defined by the user. The output typically takes the form of somatic spike times, but can in principle be any activity measurement, including somatic or dendritic voltage traces (Fig 4A). ISF then reduces the synaptic input to a minimal set of features according to a user-defined strategy and mathematical form, so that the resulting model can predict activity directly from synaptic input. Thereby, the reduced models can provide an analytically tractable description of the the input-output computation that the biologically detailed neuron model performed under the simulated *in vivo* condition (Fig 4C). This process is then repeated for diverse sets of network-embedded neuron models, covering diversity in biophysical, morphological and connectivity parameters. The analytical tractability of the reduced models enables direct comparison across reduced models to investigate how variability in neuron and network properties contributes to variability in neuronal input-output computations and consequently neuronal activity.

A further feature of the reduced models is their decreased computational expense compared to the fully detailed network-embedded neuron simulations; this enables large-scale population simulations, which nonetheless capture the effects of morphological and biophysical properties on neuronal input-output computation.

### Example application: Explaining sensory responses in barrel cortex

**Fig 5:**
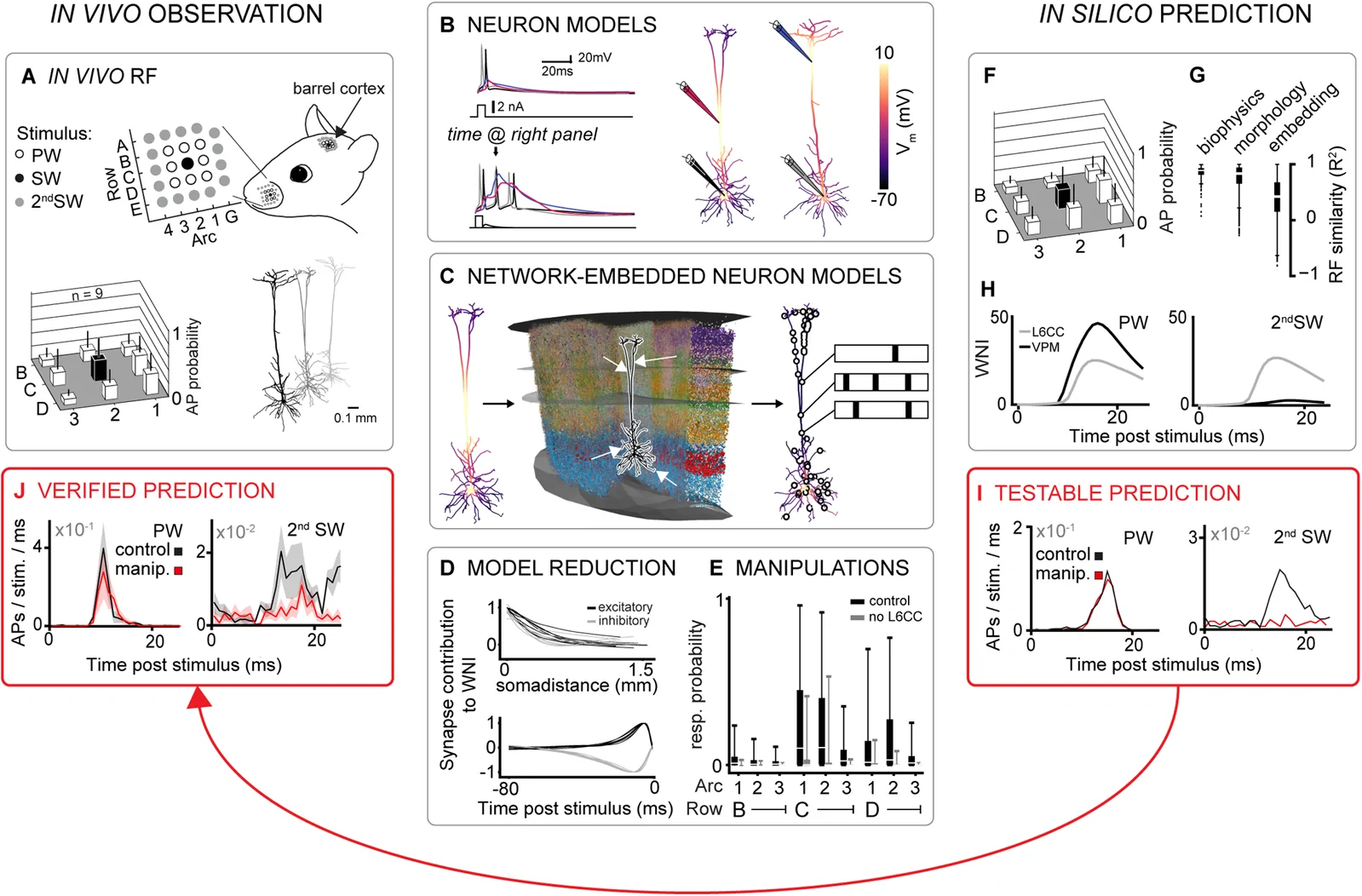
Example application of ISF to explain whisker-evoked responses in the barrel cortex. (A) *In vivo* measurements of L5PTs in barrel cortex during single whisker deflections of anesthetized rats reveal broad and heterogeneous receptive fields in neurons with morphologically diverse dendrites. Diverse L5PT morphologies are reconstructed from *in vivo* biocytin-filled neurons. Panel adapted from Bast, Fruengel *et al.* (2024) [20] (B) ISF generates multiple neuron models per L5PT morphology. These models differ in their biophysical parameters, but all reproduce the empirically observed neuronal activity. Shown on the left are the responses of two example neuron models to two current injection stimuli: a somatic current injection and a paired somatic and dendritic current injection. The right side shows the membrane voltage of two example neuron models during the paired somatic and dendritic current injection stimulus. (C) ISF embeds these neuron models into a dense model of the rat barrel cortex, and simulates the responses of these neuron models to synaptic input that mimics the *in vivo* condition of single whisker deflections in anaesthetized rats. (D) network-embedded neuron models are reduced to analytically tractable models, which weigh the contribution of individual synapses to the response probability based on location and activation time. This is the weighted net input (WNI) to the neuron, captured by spatial and temporal filters. Multiple filters are shown for different neuron models with the same morphology and network embedding. Panel adapted from Bast, Fruengel *et al.* (2024) [20] (E) Manipulations remove input from the presynaptic L6CCs. Response probability of L5PTs to 2*nd* surround whisker stimuli is abolished. (F) The *in silico* receptive fields obtained by simulating network-embedded neuron models reproduce the broad receptive fields as observed *in vivo*, while accounting for variability in neuron morphology, biophysical properties, and network input. (G) Change in receptive field (pearson *R*^2^) when changing either the biophysical properties, morphology, or the network embedding location. Changing network properties has a larger impact compared to biophysical or morphological properties. (H) Reduced models provide a mechanistic explanation by revealing how much weighted net input each presynaptic population provides to the postsynaptic neuron. Input from the thalamus (i.e., VPM) is the largest contribution for PW, but L6CC is the largest contribution for $2^{nd}SW$ responses. Panel adapted from Bast, Fruengel *et al.* (2024) [20] (I) This mechanistic insight provides a testable prediction: silencing input from L6CCs in one barrel column removes the whisker that is somatotopically aligned with the manipulated column column from the L5PT’s receptive field (manipulation, red), while leaving the responses to other whiskers unaffected (control, black). Panel adapted from Egger et al. (2020) [19] (J) This testable prediction was verified *in vivo* by abolishing L6CC input from $2^{nd}$ surround columns using muscimol injections [19]. Panel adapted from Egger *et al.* (2020) [19].

To illustrate all workflows introduced above, we show how we have applied ISF to explain the APs that the pyramidal tract neurons in layer 5 (L5PT) of the barrel cortex (reviewed in [33]) elicit in response to sensory input from different facial whiskers (Fig 5A). L5PTs integrate synaptic input from virtually all excitatory and inhibitory cell types within the barrel cortex, with large and morphologically diverse dendrites spanning all cortical layers (Fig 5A). They are the major output cell type of the cortex by sending axonal projections to various subcortical areas [34]. L5PTs elicit APs in response to whisker stimuli with onset latencies that rival those in the main input layer of the cortex: layer 4. Moreover, these fast responses occur upon stimulation of multiple different individual whiskers (Fig 5A). For decades, the origins of these fast and broadly tuned whisker responses in L5PTs were unclear, as *in vivo* experiments yielded different and conflicting conclusions [35,36]. Therefore, we decided to revisit which mechanisms account for whisker-evoked responses in L5PTs via our iterative *in silico*-*in vivo* approach [19,20].

Here, we review how we have used ISF to investigate the origins of the fast and broadly tuned whisker-evoked responses in L5PTs, including their variability across cells and trials. First, we generated neuron models of L5PTs that account for their morphology, biophysical properties and intrinsic physiology [25] (Fig 5B). Next, we embedded these neuron models into dense network models of the rat barrel cortex [24] and generated synaptic activity patterns that mimic the *in vivo* activity following passive single whisker deflection in anesthetized rats (Fig 5C). We used model reduction (Fig 5D) and targeted *in silico* manipulations (Fig 5E) to reveal that the fast onset and broad tuning of L5PTs in the rat barrel cortex are largely due to fast whisker-evoked responses in layer 6 cortico-cortical neurons (L6CCs) (Fig 5G and 5H) that are characterized by elaborate axons that project horizontally to L5PTs across the entire barrel cortex, a finding which we then tested experimentally *in vivo* [19] (Fig 5I and 5J).

#### Generating neuron models that capture the intrinsic physiology of L5PTs.

To generate neuron models of L5PTs, Bast *et al.* (2022) [25] used ISF to investigate the possible mechanisms that underlie intrinsic L5PT physiology, as characterized by backpropagating APs into the dendrites, transition to somatic burst firing upon coincident input to the soma and distal dendrites (i.e., coincidence detection [6]), and sustained firing upon sustained somatic current injection. As a result, ISF generated 4.2 million biophysically detailed multicompartmental neuron models for 12 morphologies, reconstructed from *in vivo* biocytin-labeled L5PTs. These neuron models include 35 biophysical parameters describing the conductance density of Hodgkin-Huxley type channels, *Ca*^2+^ buffering dynamics, and a scaling parameter for the apical dendrite diameter. All of these L5PT models captured the cell type-specific intrinsic physiology, but spanned a broad and diverse range of biophysical parameters, including channel expression and utilization. An explicit example of this exact workflow is provided on the documentation page of ISF [37].

#### Network-embedded neuron models of L5PTs in the barrel cortex.

For the purpose of dissecting the origins of whisker-evoked APs in L5PTs, we selected neuron models from our database that captured the morphological diversity of L5PTs and the range of biophysical parameters that could account for the intrinsic physiology of this cell type. To investigate how these neuron models integrate *in vivo*-like synaptic input, we embedded the neuron models at multiple locations of a dense neuropil model of the rat barrel cortex [27]. These embeddings provided validated predictions for the numbers and dendritic distributions of synapses from excitatory and inhibitory neurons across all layers of the barrel cortex, and from the primary thalamus of the whisker system [24]. Example code for this workflow is presented in the ISF documentation [29].

To constrain synaptic properties (strength and dynamics), we determined the peak conductance of excitatory synapses by simulating each synapse from a given presynaptic cell type onto the neuron model one by one, resulting in a distribution of uPSP amplitudes at the soma. We then scaled the peak conductance value for all synapses of the same presynaptic cell type until the cell type-specific distribution of uPSP amplitudes at the soma matched those as measured *in vitro* or *in vivo*.

For each anatomical embedding, ISF generated synapse activation patterns as we reported previously [19]. Briefly, ongoing activity was simulated by drawing individual spike times from Poisson distributions with cell type-specific mean rates fitted to *in vivo* recordings of ongoing activity [38,39]. Activity evoked by the stimulus was sampled according to cell type- and whisker-specific post-stimulus time histograms (PSTHs) following a passive single whisker deflection *in vivo* [40]. ISF can generate arbitrarily many empirically constrained activation patterns for each anatomical embedding due to the stochastic nature of (1) generating ongoing spike times by sampling from Poisson distributions, (2) generating sensory-evoked spike times from spike probabilities in PSTHs and (3) release probabilities at each individual synapse. Example code for generating synapse activity is available as an online tutorial [41].

To summarize, this workflow created a set of biologically diverse network-embedded models that capture trial-to-trial variability (i.e., activity of neurons in the network), while cell-to-cell variability was accounted for by the diversity of morphologies and biophysical parameters of the neuron models, and their embeddings into the network. Without tuning any of the parameters to the *in vivo* observation, these diverse network-embedded neuron models were able to produce *in vivo* observed single whisker responses, including response probabilities, onset latencies, and broadness, as well as the *in vivo* observed variability of these quantities across cells and trials (Fig 5 upper right). Example simulations of such network-embedded neuron models are provided in the ISF tutorials [42].

#### *In silico* predicted mechanisms and experiments.

To investigate the mechanisms underlying L5PT responses and their variations, Bast *et al.* (2024) [20] inferred generalized linear models (GLMs) from network-embedded neuron models (Fig 5D). These reduced models were optimized to determine which features of synaptic input are predictive of an AP output. Each reduced model captures the input-output computations of a single neuron model across the range of synapse activation patterns it receives during a passive single whisker deflection. The reduced models reached consensus that in order to predict whisker-evoked responses of L5PTs under this experimental condition, it is sufficient to count active synapses, weigh them by location and timing, and subtract a penalty if the neuron just fired an AP. Comparing the neuronal activity of different reduced models predicted that the *in vivo* observed variably of whisker-evoked responses across cells and trials originates predominantly from variability in synaptic input from the network, while morphology and biophysics only play a minor role (Fig 5G). An example of this workflow can be found on the ISF webpage [43].

To investigate which mechanisms underlie the broadness of L5PT responses, Egger *et al.* (2020) [19] performed *in silico* manipulations to simulated network-embedded neuron models by removing whisker-evoked input from various excitatory presynaptic cell types. While all manipulations reduced the overall AP probability, removing input from L6CCs caused the strongest reduction in surround whisker (SW) responses, and therefore caused a narrowing of the receptive field (Fig 5E). This is consistent with the finding of the reduced models, where decomposing the contribution of presynaptic cell types to whisker-evoked responses predicted that L6CCs are the main contributors to SW responses, and therefore to broad receptive fields (Fig 5H).

To provide an experimental strategy that tests if L6CCs are indeed the origin of the fast and broad responses of L5PTs, we performed additional *in silico* manipulations. In essence, these simulations predicted that deactivation of L6CCs in a single barrel column should remove the related whisker from the receptive field of any L5PT in the barrel cortex (Fig 5I). Egger *et al.* (2020) [19] performed this manipulation *in vivo* and indeed observed the *in silico* predicted outcome (Fig 5J). Taken together, our approach provided concrete and experimentally testable predictions on the neuron and network mechanisms that account for the sensory responses of L5PTs, including their variability across cells and trials.

## Discussion

The standard approach for probing the neural basis of brain functions is to record or image the activity of neurons in living animals during specific experimental conditions. Such experiments have identified neural correlates of several brain functions, ranging from sensation to perception, memory and action, and across a variety of brain areas and species. However, it remains generally unclear how the recorded cells and their underlying circuits implement these correlates. Which neurons provide synaptic input to the recorded cell, and where on its soma, dendrites or axon initial segment do these inputs arrive? Even if all this information were available, the enormous computational complexity of the dendrites will pose another challenge for inferring causality between the synaptic inputs a cell receives and it’s *in vivo* recorded somatic, dendritic or axonal activity. Thus, the mechanistic origins of *in vivo* recorded activity patterns may only be revealed if the presynaptic origins, as well as the postsynaptic locations, strengths and activation times of all inputs to a recorded cell are known, and the integration of these input patterns could be studied with respect to the complex properties of its dendrites.

Here we present our computational approaches for tackling these challenges as a fully documented and freely available software environment: In Silico Framework (ISF). ISF provides a set of workflows to generate, simulate and analyze neuron models that are well constrained by empirical observations at subcellular, cellular and network scales. In essence, ISF is the result of our two decade-long efforts to dissect cellular and circuit mechanisms of sensory processing at the cortical level [19–21,24,28] via bottom-up models of the rat barrel cortex. Like other bottom-up modeling approaches [13,14,44–46], ISF generates biophysically detailed multi-compartmental models, embeds these neuron models into anatomically detailed network models, and simulates how neuron models integrate and transform synaptic inputs from the network into action potential outputs.

It remains, however, controversial whether bottom-up models provide a viable strategy to dissect mechanistic origins of *in vivo* recorded neuronal activity, and ultimately of brain functions. Arguably, much of this criticism arises from the dilemma that current experimental techniques cannot measure all the cellular and circuit parameters that could contribute to neuronal activity under *in vivo* conditions, which is particularly true for the cerebral cortex. Consequently, despite their common goal of generating models that are well constrained by empirical data at subcellular, cellular and network scales, there are notable differences between current bottom-up modeling approaches in terms of the assumptions and the kind of empirical data they rely on, as well as the strategies and algorithms they use for integrating these empirical data to construct models. Therefore, any bottom-up model faces uncertainty on whether its assumptions are valid, whether the quantity and quality of its empirical data is sufficient, or whether the strategies and algorithms to build it are biologically plausible. Even if all relevant parameters could be measured simultaneously during *in vivo* experiments, biological variability and parameter degeneracy would still limit the generalizability of such a “perfectly” constrained bottom-up model. For example, studies have shown that biophysical model parameters generally do not transfer across dendrite morphologies, even within a cell type [25,47,48]. As it is unlikely to observe the exact same morphology twice, it remains unclear whether insights from such neuron models generalize beyond the specific neurons for which they were constructed — even in the presence of complete and perfectly accurate empirical data.

ISF was designed to address these general challenges of generating, simulating and analyzing bottom-up models. For this purpose, ISF is centered around the concept of “model consensus,” which relies on three key strategies that set ISF apart from other bottom-up modeling approaches. First, ISF enables users to generate a set of models that exhibits maximally diverse parameters, spanning the full ranges permitted by the empirically observed biological variability at subcellular, cellular and network scales. Second, ISF enables users to identify subsets of model configurations that predict the *in vivo* observations without being tuned to do so. Third, for each of the diverse model configurations, ISF enables users to identify which mechanisms at subcellular, cellular and network scales are necessary to account for the *in vivo* observations, and which mechanisms are dispensable. Thereby, ISF can determine whether mechanisms exist that are common across model configurations, and that could account for both the *in vivo* observed activity and its variability across animals, cells and trials. In essence, by achieving such model consensus, ISF predicts mechanisms that are robust despite biological variability, and which may hence be indeed utilized *in vivo*. ISF also enables users to derive model consensus from *in silico* manipulations, to identify which experimental strategies would be best suited to test the predicted mechanisms *in vivo*. Taken together, ISF provides a novel, viable strategy to dissect the mechanistic origins of *in vivo* recorded activity, which we demonstrated successfully for the barrel cortex.

### Limitations of the study

Our strategy of model consensus comes at a cost. Without tuning parameters, models generated with ISF may fail to predict the desired *in vivo* observations. One reason for such “model failure” could be that parameter diversity at subcellular, cellular or network scales is not sufficiently captured — i.e., the configurations that could predict the *in vivo* observations are simply not yet present in the set of models. Users should therefore always ensure that the set of models is sufficiently diverse, to capture both the empirically observed ranges of each parameter at each scale, as well as the diversity of possible parameter combinations across scales.

However, despite best efforts to construct models that are biologically realistic and as diverse as possible, ISF may still fail to predict a desired *in vivo* observation. For example, early on, we had generated models of a single cortical (barrel) column, which predicted *in vivo* observed responses in layers 4 [49] and 2 [18], but not of the pyramidal tract neurons in layer 5 (L5PTs) [19]. For this purpose (see also our application example), we needed to systemically quantify the substantial horizontal intracortical connectivity [50] and then incorporate these empirical data to constrain models that comprised virtually the entire barrel cortex [24]. Thus, models that assume (entirely) columnar processing, or which rely (entirely) on connectivity data from brain slices — i.e., where horizontal axons are severely truncated [24]— could, in principle, not reveal the mechanistic origins of the *in vivo* observed fast and broadly tuned responses of L5PTs. Similarly, while our models predicted the probabilities, timings, and tunings of L5PT responses, they could not predict that responses in some cells and trials occur as bursts of APs [19,20]. For this purpose, we had to incorporate our empirical observation into the models that thalamocortical axons, which provide sensory input form the whiskers to the barrel cortex, target specifically and most densely the dendritic domain of L5PTs that initiates calcium APs [21]. Model failure is hence an integral part of ISF to reveal the limits of bottom-up models and to identify experiments that could improve them. In essence, model failure addresses the questions: are the model assumptions valid (e.g., columnar processing), is the quality of the empirical data sufficient (e.g., connectivity in slices), and are the strategies and algorithms to build these models plausible (e.g., axo-dendritic overlap vs. axons target specific dendritic domains)?

Another cost of our model consensus strategy is that it requires a large set of models. Consequently, the computational resources that ISF requires for generating, simulating and analyzing these models can be enormous, as we estimate in the Supplement for our application example in the barrel cortex (S1 Table). We hence designed ISF for high-performance compute infrastructures. However, the computational cost depends on the complexity of both the models and the *in vivo* observations under investigation, and may be substantially less compared to our use-case, where neuron models needed to capture the highly complex dendritic physiology of L5PTs, as well as inputs from neurons across the entire barrel cortex.

Our model consensus strategy could also set the stage to investigate the mechanistic origins of *in vivo* observed network dynamics at unprecedented levels of detail. However, we did not yet implement a workflow within ISF to simulate recurrent dynamics in networks of biophysically detailed neuron models. Even so, there is no principle reason why ISF could not be used for simulations of recurrent network dynamics. This would require to generate sets of network models where both the network configurations and their constituent biophysically detailed neuron models capture the empirically observed ranges of each parameter at each scale, as well as the diversity of possible parameter combinations across scales. Simulating and analyzing such sets of biologically diverse neuron-network models, and achieving model consensus about the mechanistic origins of *in vivo* observed network dynamics, will however require enormous computational resources. Incorporating advances in simulation technology (e.g., Jaxley [51]) into ISF may provide the necessary means to overcome these computational challenges by scaling to large GPU infrastructures.

In addition, our approaches in ISF to reduce the biophysically detailed neuron models to analytically tractable models, as well as other model reduction approaches [52], may provide the means to simulate recurrent dynamics in networks with similar biological realism, but at substantially lower computational costs. Well-established frameworks for simulating network dynamics such as NEST [53] and The Virtual Brain [54] indeed provide strategies for incorporating more biological details into network models of integrate-and-fire point neuron models, e.g., by constraining connection probabilities based on data from brain slices. Incorporating such biological details generally impacts the predicted dynamics of such simplified network models significantly. For example, by incorporating connectivity measurements from the barrel cortex into such models, we showed how empirically observed diversity in the number incoming synaptic inputs per neuron impacts the dynamical balance between excitation and inhibition in layer 4 [55]. Thus, replacing integrate-and-fire point neuron models with those generated by ISF via model reduction, may be a way to investigate network dynamics at increasing levels of biological realism. By making our code available and well-documented [56]  we hence seek to facilitate collaborative efforts that combine ISF with other frameworks [46,52,53,57] to simulate cortical dynamics at unprecedented levels of detail.

### Outlook

Dissecting the cellular and circuit mechanisms that underlie *in vivo* recorded neuronal activity remains a major challenge. Current experimental techniques are insufficient to simultaneously measure all relevant variables under *in vivo* conditions. However, our vision for the rodent whisker system to dissect the mechanistic origins of its functions by generating, simulating and analyzing anatomically and functionally realistic models is shared broadly across the neurosciences. For example, large-scale initiatives, such as the Retinal Functomics consortium or the Allen Institute for Brain Science combine functional imaging of neuronal activity in the mouse retina [58,59] or visual cortex [60,61] with dense EM reconstructions. Similar experimental efforts are made for fruit flies, where an entire brain has already been reconstructed at EM resolution [62–65]. Thus, the empirical data that is necessary to generate bottom-up models similar to those that we developed for the barrel cortex is becoming increasingly available. ISF may hence be readily applicable to a growing number of model systems.

In summary, ISF’s key concepts are (1) generating sets of empirically well-constrained models that capture biological diversity and degeneracy within and across subcellular, cellular and network scales, (2) simulating *in vivo* conditions without parameter tuning, (3) analyzing the simulations to achieve model consensus about mechanistic origins that could account for *in vivo* recorded activity and its variability across animals, cells and trials, and (4) using *in silico* manipulations to identify experimental strategies that would be best suited to test whether the predicted mechanisms are indeed utilized *in vivo*. While we demonstrate the value of ISF for the barrel cortex with empirical data that is accessible today, we believe that the power of ISF and of its underlying concepts will continue to increase as new experimental techniques become available. For this reason, we make ISF available as a fully documented online resource that will facilitate iterative *in silico* – *in vivo* investigations into the mechanistic origins of *in vivo* functions.

## Methods

### Ethics statement

No animal experiments were carried out in this study. The previously reported animal experiments [40], were carried out after evaluation and approval by the local German authorities, and in accordance with the animal welfare guidelines of the Max Planck Society.

### Implementation of in silico framework

In this section, we provide a detailed overview of how ISF implements the aforementioned workflows, as well as a general implementation overview. A high-level dependency graph and an overview of the most important file formats used in ISF are given in S1 and S2 Figs. The online documentation provides a complete API reference [22], in addition to tutorials [23] for the workflows outlined in the Results, and a specification on all file formats used in ISF [66]. We highlight some key features below and refer to the corresponding sections of the API reference.

ISF is written entirely in Python 3, with the exception of the NEURON mechanisms for channels and synapses, which are written in NMODL. It uses the NEURON simulation environment [67] as a backend, and provides extensive parallellization capabilities with Dask [68]: a mature and widely adopted multiprocessing library. Installation and environment management are handled through pixi [69], providing version-controlled environment lock files to ensure reproducibility. ISF is available for Linux, macOS and Windows (Windows support is experimental) and only requires minimal dependencies: pixi and git. Windows additionally requires a user-based installation of NEURON. Installation instructions for all OS’s are available online [70]. ISF is open-source under Apache 2.0, subject to continuous integration testing, and fully documented [22]. In addition, we provide tutorials [23] where we apply the workflows from the Results on L5PTs in the barrel cortex.

The Interface package provides top-level API access to all important workflows and classes, often only requiring a single function call to start a workflow. It is recommended to use ISF through Interface:

import Interface as I

I.simrun_run_new_simulations(...) # e.g.

### Neuron modeling

A neuron model in ISF is defined by a .hoc morphology file, and a single parameter file that contains passive and active properties in the .param format [26]. The single_cell_parser package provides convenient API to build multi-compartmental neuron models for simulation in NEURON.

Generating neuron models in ISF consists of two steps: optimization to find a seed model whose responses to current injection stimuli are consistent with empirical physiology, and then exploration to discover the full parameter space (i.e., degeneracy) of models consistent with empirical physiology. The empirical data this workflow requires as an input are (1) a morphology reconstruction, in .hoc format, (2) electrophysiological recordings that capture the neuron’s intrinsic physiology, and (3) bounds on known biophysical parameters (e.g., ion channel distributions) which describe the neuron’s passive and active properties, including intracellular dynamics if applicable. The electrophysiological recordings are parametrized into separate attributes (such as AP height and width, spike frequency etc.), hereafter called “objectives.” Given the empirically observed diversity of neuronal activity, each objective may be specified as a distribution.

The optimizer currently used in ISF is BluePyOpt’s [71] implementation of an indicator-based evolutionary algorithm (IBEA) [72]. Each iteration, BluePyOpt generates new sets of biophysical parameters, simulates a set of stimuli to each neuron model and evaluates the resulting responses to current injection stimuli by comparing the simulated result to each empirical objective. Neuron models that cannot be compared to a given objective (e.g., because they have no AP at all) are given a penalty value. The neuron models that had the best evaluation are then used to construct new models in the next “generation,” with some stochasticity. This continues until a model is found which is empirically consistent, meaning all attributes of its responses lie in-distribution of the empirically observed objectives.

ISF’s exploration algorithm [25] relies on the availability of a valid “seed” model that captures the desired empirically observed intrinsic physiology. For our example of L5PTs, BluePyOpt [71] was most successful in identifying seed models that captured the complex dendritic and perisomatic physiology of this neuronal cell type. However, any method that provides one or more seed points is compatible with ISF, including manual tuning, randomly sampling parameters (e.g., as in Roy and Narayanan (2023) [73]), gradient-based optimization (e.g., with Jaxley [51]), and recent extensions and frameworks around BluePyOpt [74]. The exploration algorithm then makes small random variations on these biophysical parameters, simulates the same stimuli and checks if the responses are still in-distribution of the empirically observed objectives. For all newly generated models that pass this check, this process is repeated. The direction of variation in the biophysical parameters can be configured to favor various metrics such as variance, increasing euclidean distance in parameter space, novelty, or others. In our experience, random variations were sufficient to explore a broad diversity of neuron models per morphology. A common stopping criterion for this exploration is to keep track of the variance of all found neuron models, and interrupt the exploration once the marginal increase in variance fails to reach a user-defined limit.

These routines are implemented in the biophysics_fitting package, which allows the user to configure any set of repeatable stimulus and evaluation protocols, and vary the biophysical parameters as a single parameter vector. Any parameter listed in ISF’s neuron parameter specification [26] can be modified, and hence used during optimization and exploration. Specifically, the ISF neuron parameter specification includes (1) ISF-specific parameters for spatial profiles of channel densities, (2) all NEURON parameters defined in the PARAMETER block of a loaded NMODL file, and (3) parameters used for ISF-specific cell modification functions, defined in the single_cell_parser.cell_modify_functions module. NEURON parameters for ion channels typically only include the conductance densities of ion channels for a given compartment of the neuron model. For calcium buffers, these are typically the parameters of the diffusion kinetics, such as the time constant, shell depth, and ratio of unbuffered *Ca*^2+^. However, any parameter of a NEURON mechanism can in principle be exposed by defining them in the PARAMETER block of the corresponding NMODL file, including channel opening and closing rates, temperature scaling coefficients, or calcium buffering and extrusion rates. In addition, the custom functions under single_cell_parser.cell_modify_functions can be applied to a neuron model by listing the function name, and their keyword argument names and values in the neuron parameter file. These parameters are explained in detail on our documentation page [66].

Both the optimization and exploration algorithm can be started from Interface:

biophys_empirical_range = DataFrame([min_values, max_values])

# optimize

emp_consistent_neuron_models = I.bfit_start_run(biophys_empirical_range)

# explore

I.RW(...).run_RW(emp_consistent_neuron_models, biophys_empirical_range)

The tutorials provide explicit examples on how to use these packages for simulation [75], model evaluation [76], and model generation [37].

### Network-embedded neuron models

The singlecell_input_mapper [30] package provides all functionality to generate anatomical network embeddings for a given morphology as described in Udvary *et. al.* (2022) [24] under singlecell_input_mapper.udvary2022. It is provided as a stand-alone package to allow the addition of other network embedding methods in the future. The purpose of any current or future network embedding method is to generate the synapse locations and presynaptic origin for a neuron morphology in the form of .syn [31] and .con [32] files. Alternatively, users can define these files themselves, e.g., based on dense EM reconstructions.

The input to the network embedding workflow is a model or reconstruction of the dense neuropil populated with annotated neuron morphologies, i.e., the cell ID of each neuron and their associated cell type, dendrite and axon reconstructions, the number and locations of pre- and postsynaptic structures (e.g., dendritic spines and axonal boutons), and the morphology of the biophysically detailed neuron model to be embedded (hereafter referred to as postsynaptic). Our dense neuropil model of the rat barrel cortex has been described and validated previously [24], can be downloaded and explored interactively [77], and can now additionally be downloaded via the pixi front-end in ISF:

$ pixi run download_bc_model

Briefly, singlecell_input_mapper.udvary2022 discretizes the neuropil into voxels of a user-specified size, and identifies where the postsynaptic morphology intersects with these voxels. Synaptic connections are only possible if pre- and postsynaptic structures are located in the same voxel. For each of these voxels, synapses are created onto the postsynaptic neuron model from presynaptic neurons in the dense neuropil model/reconstruction based on their respective pre- and postsynaptic structures in the voxel and user-defined synaptic wiring rules [24,28]. The anatomical network embeddings are saved in the form of two files. A synapse distribution file (.syn [31]) describes where each synapse connects onto the postsynaptic morphology. A synapse connectivity file (.con [32]) describes to which presynaptic cell each synapse belongs, and which cell type this is. Any dense EM reconstruction is compatible with ISF as long as its description of connectivity onto the postsynaptic cell can be translated to these .syn and .con files. More information on this workflow can be found in the associated tutorial [29].

In order to generate synaptic activity onto the postsynaptic neuron, singlecell_input_mapper [78] converts all presynaptic cells to point neurons that emit spikes according to cell type-specific activity recorded during the *in vivo* condition to be explained. ISF provides functionality to define activity with different distributions, such as Poisson (default), normal, uniform, log-normal, or exact activations. ISF generates activation times at each individual synapse on the postsynaptic neuron based on its release probability, which can be defined for each presynaptic cell type individually. Each presynaptic cell type’s synapses are parameterized by delay, weight and dynamical parameters such as rise and decay time constants, and could include additional empirically constrained parameters, e.g., to capture different plasticity mechanisms [19,21]. Explicit code examples on how to perform this workflow is shown in the associated tutorial [41].

A single network model is captured by one parameter file [79], containing information on the anatomy (references to .syn [31] and .con [32] files), cell type-specific activation probabilities for an *in vivo* stimulus, synapse dynamics, and synaptic weights. It is important to note that two of these parameters are stochastic: the spike probability of presynaptic cells, based on their respective distributions, and the synapse activation probability upon a presynaptic spike. Therefore, each parameter configuration can be used to generate a set of synapse activation patterns, capturing trial-to-trial variability.

### Simulating network-embedded neuron models

The simrun package simulates network-embedded neuron models from neuron and network parameter files using NEURON as a simulation engine. A tutorial for this process is available online [80]. It expects both parameter files, and can run multiple trials per neuron-network parameter configuration to capture trial-to-trial variability in cell and synapse activation patterns. These trials are parallelized across processes by default. A full workflow can be started with a single function call:

delayeds = I.simrun_run_new_simulations(

neuron_parameters,

network_parameters,

output_path,

n_trials,

) # returns delayed functions for parallel execution

distributed_scheduler.submit(delayeds) # e.g. using a Dask scheduler

The simulation results can be efficiently parsed and stored by the data_base package. By default, these parsed simulation results include the membrane voltage of the neuron model at the soma and other specified recording sites, the exact cell and synapse activations that were used for that trial, and a copy of the parameter files that were used to generate these results for each simulated trial [81]. Converting the raw simulation output to high-performance dask dataframes in the msgpack or parquet format can be done by:

db = I.DataBase("db_path")

I.db_init_simrun_general(db, simrun_results_path)

### Manipulations

Re-running existing simulations with various manipulations to the neuron, network and synapses is achieved as follows:

I.simrun_rerun_db(

db,

neuron_param_modify_functions=[scale_diameter],

network_param_modify_functions=[decrease_ampa_tau1],

synapse_activation_modify_functions=[add_activations],

)

Manipulations are defined by functions that accept a neuron parameter file, network parameter file, or synapse activation dataframe, and return it changed. This allows to re-run existing simulation trials (or a subset thereof) while making targeted manipulations, such as (but not limited to): scaling synaptic weights by distance, receptor type, presynaptic origin, or individually; changing synaptic activation patterns by the same categories; removing connectivity to presynaptic cells or populations; changing biophysical parameters; any combination of the above. A specific example that silences synaptic activations of a specific presynaptic population while keeping all other synapses exactly the same is provided in the online tutorials [42], which replicates the same manipulations as described in Egger *et al.* (2020) [19] on a small sample size.

### Reduced models

The goal of model reduction is to capture the relationship between synaptic input and neuronal output in simulated network-embedded neuron models with a minimally sufficient description. A reduced model is a function *f* that predicts the neuronal output *o* from synaptic input 𝐃, based on tunable parameters 𝐱:


$$\begin{array}{c} f:𝐃,𝐱\to o \end{array}$$ (1)

To build reduced models, ISF provides three modular components under simrun.modular_reduced_model_inference: a DataExtractor, Strategy, and Solver. These components define 𝐃 and *o*, *f*, and 𝐱 respectively. Each component can be configured by the user to best address their research question, depending on the *in vivo* condition, neuron models and network models under investigation. First, the DataExtractor allows the user to define data processing workflows that extract relevant features from the input and output of fully detailed simulated network-embedded neuron models. This defines 𝐃 and *o* respectively. For example, the output commonly takes the form of somatic spike times, but any aspect of the neuronal output can in principle be used, including somatic voltage traces, burst occurences, or dendritic events. Second, the Strategy object allows the user to define the exact mathematical form of the inference function *f*, including tunable parameters 𝐱. ISF provides predefined Strategy objects, such as Generalized Linear Models (GLM) and Linear Discriminant Analysis (LDA) classifiers. Third, the Solver object defines a cost function g:o→c and an optimization algorithm. The cost function *g* captures how well the output *o* produced by the inference function matches the actual neuronal output, e.g., a simple mean squared error (MSE) or the area under the receiver-operator curve (AUROC). The Solver object then adapts 𝐱 according to the user-defined optimization algorithm, e.g., COBYLA [82].

As a concrete example application, we provide an online tutorial [43] reproducing the results from Bast, Fruengel *et al.* (2024) [20]. Here, the DataExtractor extracted the soma distance and activation time of each synapse as input data 𝐃, and the AP time points as output *o*. Next, the Strategy object defined *f* as a Generalized Linear Model (GLM), whose linear component were filter functions that weigh the contribution of each synaptic activation to the neuronal output *o*, depending on their soma distance and activation time. The filter functions were linear combinations of raised cosine basis functions, and the tunable parameters 𝐱 were defined as the weights of this linear combination. The nonlinear component of the GLM assigned a penalty to recent APs, to capture refractoriness. Finally, the Solver object defined the cost function as the AUROC of the AP response probability, and defined COBYLA [82] as the optimization algorithm to adapt the weights 𝐱. Each reduced model was generated for one particular neuron model, embedded at multiple locations to capture network variability. The resulting reduced models were able to predict the AP response probability based on the soma distance and activation times of each synapse. The tuned filter functions of these reduced models allowed an analytically tractable comparison for how different neuron models weigh synapse activations, across a wide diversity of input patterns. Inference with these reduced models sidestep simulating compartmental neuron models, but the synapse locations (or at least their distance to the soma) and activation times need to be known, which still requires the network embedding workflow to be run. Each component used in the creation of this particular reduced model — i.e., the spatiotemporal synapse data extractor, raised cosine basis functions, wrapper to the COBYLA optimizer, and cost function — is available in ISF as predefined components.

### Data management

ISF implements an intuitive data_base package with modern and performant data formats, such as parquet [83] and zarr [84], as well as an updated pandas interface to the msgpack format using fast and efficient compression such as blosc [85,86]. Extra care is taken into account to ensure all data is: (1) reproducible, (2) can be written or converted to standard file formats, and (3) saved efficiently, both in terms of IO speed and file size. To ensure reproducibility, all data saved with ISF’s data_base package contains rich metadata in human-readable JSON format. This metadata includes the commit hash of the code used to generate the data, creation date, file format, and which reader to use in order to load in the data. In case the data was generated in an interactive IPython session, such as Jupyter notebooks, data_base also saves a history of the IPython code as metadata using the %history magic command.

For high performance computing (HPC) contexts, ISF provides compatibility with multiple file locking servers: Apache Zookeeper, Redis, and simple file-based locking with fasteners. In addition, data_base is easy to use thanks to the Python dictionary-like syntax:

db = I.DataBase("path/to/data")      # initialize database

db["saved_data"] = my_data         # infer file format & save

loaded_data  =  db["saved_data"]      # load data back in memory

# specify a format

from data_base.IO.LoaderDumper import dask_to_msgpack

db.set("data_in_msgpack", my_data, dumper = dask_to_msgpack)

These considerations ensure high and stable performance, as well as data portability without the need for an additional interface or specialized data format.

### Visualization

ISF provides extensive visualization capabilities for neuron models and network-embedded neuron models. Individual frames or videos of neuron models and network-embedded neuron models can visualize their membrane voltage, ion currents, and synapse activation patterns. The three outputs of the example code below is shown in S3 Fig.

from visualize.cell_morphology_visualizer import CellMorphologyVisualizer

cmv = CellMorphologyVisualizer(cell)

cmv.plot(color="black")     # A: plot morphology

cmv.plot(

color = “Ca_HVA.ica”,    # B: plot Ca_HVA current

time_point = 1034,      # at some time point

highlight_section = 73,    # use an arrow to highlight section 73

highlight_x=0.9        # ... at relative co x=0.9

)

cmv.animation(          # C: Create video

color = “voltage”,      # show the voltage

show_synapses = True      # show the synapse activations

)

These visualizations can be exported to VTK for postprocessing in VTK-compatible software suites like ParaView [87]. ISF additionally includes BlenderSpike [88] as a dependency for postprocessing in Blender. The combination of the two can create high-quality figures and animations. An example of both is shown in S4 Fig.

### Stability and extensibility

ISF is designed such that it can integrate future work, without breaking existing functionality. ISF takes several measures to facilitate this goal.

First, ISF explicitly defines a stable API boundary in a dedicated interface, aptly called Interface. By convention, any function or class exposed in the Interface module may not change its API or functionality. Extensions can be made by adding keyword arguments to functions, whose default value should maintain the previous functionality. Classes can be extended with new methods and attributes. In case of incompatibilities it is preferred to create a new class or function with a new name, while keeping the previous function accessible and unchanged.

Second, the data_base module separates how data is saved (i.e., the data format) from the syntax on how to load it. This design makes it is possible to change the data format post hoc, should a better format become available. The data remains accessible via unchanged syntax. Thereby, previous code, including data analysis notebooks, do not need to be modified when updating data formats. New data formats can be easily added by adding a module to the data_base.IO.LoaderDumper package, which only needs to implement a set and get method. Thanks to this convention, simulation data that we saved more than a decade ago (in Python 2) is still readable with ISF today.

Third, we provide a single python environment to run all functionality within ISF. This minimizes the need to set up project specific environments and facilitates the exchange of code and data across projects and lab members. This python environment is version-tracked thanks to pixi’s lockfile capabilities. As an additional precaution, the git commit is saved in the metadata of each data object, providing a link to the exact Python environment that was used to save the data.

## Supporting information

S1 File Computational costs. (PDF)

S1 Table Stimulus protocols for capturing L5PT intrinsic physiology [6,47] and their respective runtimes for simulations with ISF and NEURON [67]. Neuron models had on average 852.00 compartments (*STD* = 172.28).
(PDF)

S1 Fig Dependency graph of ISF. Interface provides direct and high-level API access to core workflows in ISF. single_cell_parser creates morphologies, builds and provides API access to biophysically detailed neuron models, and uses NEURON in the backend to simulate these. biophysics_fitting uses single_cell_parser to optimize biophysical parameters and additionally explore diverse neuron models. singlecell_input_mapper infers synapse locations onto a neuron morphology based on a dense connectome model, as well as generating synaptic activity from empirical data. simrun provides high-level API access to build and run network-embedded neuron models, and reduce these to analytically tractable representations. Results are saved using data_base, additionally allowing to re-run existing simulations with manipulations. In addition to the core packages shown above, ISF also includes tests, docs, config, installation and tutorial material (installer, getting_started), and analysis tools (spike_analysis and visualize). All source code is available on our public repository, accessible via our homepage [56].
(TIF)

S2 Fig Overview of parameter files in ISF. ISF packages are shown in pink with two snipped corners. Parameter files are shown in white with rounded corners. Morphology files are used by biophysics_fitting to generate a dataframe of possible biophysical parameters, each row defining a single empirically constrained neuron model for this morphology. These are then parsed into neuron parameters by single_cell_parser. The same morphology file is used by singlecell_input_mapper to create network embeddings, each of which defined by a single .syn file and .con file. The former to define synapse locations onto the morphology, the latter to define their presynaptic origin. Together with activity data, they are parsed into network parameters. The simrun package takes these neuron and network parameters to run simulations of network-embedded neuron models.
(TIF)

S3 FigThree outputs of ISF’s visualize module.(A) The morphology in black. (B) Transmembrane current flowing through $Ca_{HVA}$ channels at a fixed time point *t*. An arrow highlights section 73 at relative coordinate *x* = 0.9. By default, only dendrites that contain this channel are visualized. (C) A stillframe of an animation at the same time point *t*, showing the membrane voltage and synapses active (spheres, different colors represent different presynaptic cell types) between *t* and *t* + 1*ms*.(TIF)

S4 Fig Left: The soma locations of presynaptic cells that are active in [*t*, *t* + 1*ms*], and the simulated neuron model color-coded with the membrane potential for a given 1*ms* time interval. Surfaces of pia (top) and white matter (bottom) shown in off-white. All components of this plot were exported to VTK using ISF and visualized in ParaView [87]. Right: The same simulation visualized in Blender. Components are again exported to VTK using ISF, except for the neuron model in the middle, which is exported using BlenderSpike [88]. A sample of L5PT morphologies surround the neuron model (1 morphology in each column of the barrel cortex). Membrane voltage color-coded and set to an emissive surface.
(TIF)
