# Supplementary material for: An in silico framework for dissecting the mechanistic origins of in vivo recorded neuronal activity
Source: PLoS Comput Biol. 2026 Aug 21;22(8):e1014617. doi: 10.1371/journal.pcbi.1014617 (PMC13529221; doi:10.1371/journal.pcbi.1014617)
Supplement: Supplementary file 1 [file pcbi.1014617.s001.pdf]

Supplement

Computational cost

Below, we report the computational cost for our own use cases with ISF: explaining whisker-evoked responses of layer 5 pyramidal tract neurons (L5PTs) in the rat barrel cortex. Reported values were measured on `Intel(R) Xeon(R) Gold 6138 CPU @ 2.00GHz` and a GPFS filesystem on a `Broadcom/LSI MegaRAID Tri-Mode SAS3408 RAID controller (PCIe, 33MHz)` operating `66 MHz SATA` hard drives.

The computational cost of generating neuron models is dominated by the cost of simulating each current injection stimulus. S1 Table reports the runtimes for the stimulus protocols we have used for our modeling approaches [6,47]. These stimuli are used during both optimization and exploration. During exploration, it is recommended to implement an early stopping criterion. For example, when either of the first two (shorter) stimuli produced unsatisfactory results [47]. The subsequent, consequent (longer) stimuli can be skipped, since we already know the neuron model is inconsistent. This significantly increases the speed of exploration. As previously reported, to investigate the diversity of neuron models, we have generated ~ 15.5 million models for 12 L5PT morphologies [25].

Embedding neuron models into network models took, on average, 84.56 seconds per embedding for the methods described in Udvary *et al.* (2022) [24]. The workflow setup took between 6 and 14 minutes, dominated by reading in axon tracing data, which was in our case ~ 18000 files that each took $\sim 20 - 30ms$ to read. Memory usage was $\sim 4.0GB$ above baseline per process that performed an embedding, mostly dominated by storing bouton data ($\sim 2.1GB$) and storing a 3D synapse density field ($\sim 1.8GB$). These values are highly dependent on the size of the dense neuropil model. To capture network variability in the barrel cortex, we generated 50 embeddings per embedding location, per morphology, and took the embedding closest to the distribution mean as a representative network embedding for further simulation [19]. Bast *et al.* (2024) [20] embedded 5 morphologies across 81 locations, yielding $5 \times 81 \times 50 = 20250$ embeddings; Egger *et al.* (2020) [19] embedded 1 morphology across 9 locations, yielding 450 embeddings.

The ratio of wall time to simulation time for network-embedded neuron models during *in vivo*-like synaptic input conditions was on average 79.35 ($SD = 11.48$). E.g., a process simulating 3 seconds of activity ran ~ 4 minutes on our hardware. To capture variability in synaptic input, we typically simulate $10 - 100$ trials per neuron model, per embedding location.

It is important to note that these values were measured on HPC hardware. It is highly likely that our `GPFS RAID` filesystem is slower than common domestic SSD's during the IO-bound setup of the synapse mapping method, and while domestic machines have less available CPUs for parallelization, it is not unlikely that on a core-by-core basis, well-performing domestic CPU may outperform our `Intel Xeon Gold` for simulating neuron models or network-embedded neuron models. In addition, our examples require that the neuron models capture the complex dendritic physiology of L5PTs, and that the network models capture synaptic inputs from neurons across the barrel cortex. Thus, neuron models with less complex physiology and with synaptic inputs from networks smaller than an entire cortical area likely require less resources, and may even be feasible without access to large-scale computational infrastructure.
