# Supplementary material for: An in silico framework for dissecting the mechanistic origins of in vivo recorded neuronal activity
Source: PLoS Comput Biol. 2026 Aug 21;22(8):e1014617. doi: 10.1371/journal.pcbi.1014617 (PMC13529221; doi:10.1371/journal.pcbi.1014617)
Supplement: Supplementary file 2 [file pcbi.1014617.s002.pdf]

	Stimulus				Simulation	
Name	Shape	Site	Amplitude (nA)	Duration (ms)	Duration (ms)	Wall time (s)
bAP	Step	Soma	1.9	5	600	4.89 ± 0.59
BAC	Step	Soma	1.9	5	600	6.30 ± 0.55
	EPSP	Apical Dendrite	0.5	τ_1=1.0 τ_2=5.0	3000	
Step 1	Step	Soma	0.619	2000	3000	59.17 ± 13.61
Step 2	Step	Soma	0.793	2000	3000	73.31 ± 5.70
Step 3	Step	Soma	1.507	2000	3000	97.28 ± 13.74
