# Supplementary material for: An in silico framework for dissecting the mechanistic origins of in vivo recorded neuronal activity
Source: PLoS Comput Biol. 2026 Aug 21;22(8):e1014617. doi: 10.1371/journal.pcbi.1014617 (PMC13529221; doi:10.1371/journal.pcbi.1014617)
Supplement: Supplementary file 3 [file pcbi.1014617.s007.pdf]

Reviewer #1:

This manuscript presents the In Silico Framework (ISF), an open-source environment for generating and analyzing ensembles of biologically detailed neuron-network models. The central conceptual contribution, shifting the focus from single "tuned" models to diverse ensembles that capture biological degeneracy, is a timely and significant step for computational neuroscience. The integration of multi-scale data to achieve "model consensus" is a powerful approach for making testable in vivo predictions. The framework is technically impressive and well-demonstrated through its application to the rat barrel cortex. I support publication, as ISF aligns perfectly with the scope of PLOS Computational Biology. However, the manuscript would benefit from several specific clarifications to help potential users understand the practical deployment and limitations of the tool.

We thank the reviewer for the very positive and constructive feedback, and for supporting the publication of our manuscript in PLOS Computational Biology. We rewrote several parts of the manuscript to help users to better understand the practical deployment and limitations of ISF. Moreover, we substantially revised the source code and online tutorials to facilitate that ISF is readily applicable to other models and model systems – i.e., we now improved the separation of core functionalities of ISF (e.g. embedding of neuron models into network models) from our application examples in the barrel cortex. We provide detailed responses to all your suggestions below, and highlighted all changes in the revised manuscript in yellow.

Minor 1: Data Requirements & Accessibility. While the manuscript notes that statistical methods can be used to generate neuropil models as an alternative to dense EM reconstructions, many researchers may lack such resources or pre-established statistical models. Could the authors more explicitly define the "minimum viable data" required to use ISF? Even a short schematic outlining required versus optional inputs would greatly increase accessibility for potential adopters.

*We agree with the reviewer that generating dense neuropil models, constrained by (or directly derived from) dense EM data, is challenging. Therefore, the online resource now describes more clearly how to generate such neuropil models. More specifically, we now specify the anatomical data that was necessary to generate the dense barrel cortex models (**link**), we added a tutorial that explains the contents and formats of these anatomical data (**link**), linked this tutorial to the main tutorial for network embedding (**link**), and we extended the online data format overview with a toggle that specifies the sources and kinds of these anatomical data (**link**). However, while we emphasize the benefit of embedding neuron models into neuropil models **(lines 131-144)**, we now also state in the manuscript **(lines 145-151)** and throughout the online resource (e.g. **link**) how ISF could be used by user-defined network embedding strategies – i.e., as long as our data formats are met, the minimum viable data to use ISF are specifications of synapse numbers and locations along morphologies of neuron models, and of the respective cells from which these synapses originate in a network model. The revised code of the network embedding procedure (**link**) hence provides our strategy for the barrel cortex (i.e., from Udvary et al.,) now as a separate package, leaving the option to include other user-defined strategies. **In sum**, the network embedding strategy is left to the user, while we encourage and explain how to generate and use a dense neuropil model.*

Minor 2: Computational Cost. To help the community assess the feasibility of adopting ISF, it would be helpful to provide approximate estimates of computational requirements (e.g., CPU-hours, peak memory, and storage) for a representative workflow. Even order-of-magnitude estimates would be informative for groups without access to large-scale computational infrastructure.

*In the supplement, we now provide information on computational costs for our example of simulating whisker-evoked responses in the barrel cortex (new paragraph "**Computational Cost**" and new **Table S1**), and we added a statement to the Discussion **(lines 476-480)** that computational costs will depend on the complexity of the models and research questions.*

Minor 3: Troubleshooting "Model Failure". The authors state that if no model reproduces an in vivo observation, the generation process requires further refinement. This is a critical juncture for any user. Could the authors expand on the recommended diagnostic workflow in such cases? For instance, how might a researcher systematically distinguish between a missing biological mechanism versus an insufficiently explored parameter space or overly restrictive empirical constraints? Providing guidance on this would strengthen the methodological transparency of the framework.

We thank the reviewer for bringing up this issue. "Model failure" is indeed an integral part of ISF to reveal the limits of the models and to identify experiments that could improve them. In a new Discussion paragraph, we now highlight this importance of model failure, as it is a direct consequence of ISF's concept to achieve "model consensus" without parameter tuning, and we give guidance from our own experience on how to address model failure ***(lines 450-471)****.*

Minor 4: Mathematical Form of Reduced Models. The model reduction section remains somewhat abstract. While GLMs are mentioned in the example application, it is unclear what "minimal set of parameters" means in the general case. Does ISF provide a library of reduction methods (e.g., GLMs, kernel-based approaches, specific filter architectures), or is the user expected to define the mathematical form entirely? A clearer description of the interface for implementing reduced models, and what is provided out of the box, would significantly improve clarity in this important conceptual component of the framework.

We agree with the reviewer that the model reduction section needed improvement. In the revised manuscript, we now introduce our concept of model reduction for specific in vivo conditions ***(lines 228-241)*** *and describe our approaches formally* ***(lines 723-743)****. In brief, ISF enables model reduction by providing sets of utility functions across three modules. (1) The data_extractor module enables users to specify which synaptic input features a reduced model should relate to which neuronal output features. (2) The strategy module enables users to define the mathematical forms of the inference functions that predict neuronal output from input. ISF provides example Strategy objects, such as GLMs, including a selection of basis functions (e.g. raised cosine). (3) The solver module enables users to define cost functions that capture how well the output produced by a reduced model matches the output of the multi-compartmental model (e.g. mean squared error or area under the receiver-operator curve). Furthermore, the solver module enables users to use built-in (or their own) optimization algorithms to identify reduced models that predict the full model output best. We describe how we had used the three modules for our example of L5PT responses in the barrel cortex* ***(lines 744-766)****, and provide it as an online tutorial* ***(link).***

Overall, I believe this manuscript represents a valuable and timely contribution. With clarification of the above points, it will serve as an important resource for researchers interested in ensemble-based, mechanistic modeling of in vivo neural activity.

We thank the reviewer for this positive conclusion.

Reviewer #2:
The manuscript by Meulemeester et al. introduces the In-Silico Framework (ISF) for iterative investigation of single neuron function, bridging in silico predictions with in vivo observations. The proposed framework is a valuable contribution to the field, as it offers a way to integrate the expanding body of experimental data to advance mechanistic understanding of neural activity in vivo. The authors provide compelling examples of the framework's application based on their prior research. However, the generalizability of the framework is not immediately clear from the manuscript or the provided code. That said, the manuscript is well-written and visually engaging, with clear illustrations. The accompanying code is publicly available, well-documented, and includes helpful tutorials. Positive aspects: 1. The proposed workflow effectively bridges in silico simulations and in vivo observations, demonstrating a well-structured and systematic approach. It incorporates rigorous sensitivity analysis and parameter exploration, enabling targeted manipulations and identifying key mechanisms of neuronal computations. 2. The provided examples are highly compelling and provide solid evidence of the predictive power of the framework. 3. The visuals produced by the package are of high quality. 4. Performance considerations are addressed by the use of scalable tools such as Dask, ensuring efficiency and applicability to large datasets.

We thank the reviewer for the thorough review and constructive feedback. We rewrote several parts of the manuscript and substantially revised the code and online tutorials to facilitate that ISF is more generally applicable to other models or model systems. For example, as suggested by the reviewer, we now made the source code more configurable and less reliant on hardcoded data, and separate core functionalities of ISF (e.g. embedding of neuron into network models) from barrel cortex examples. We provide detailed responses to all your suggestions below, and highlighted all changes in the revised manuscript in yellow.

Major 1. I recommend balancing the content of the Introduction and Discussion sections. The authors might follow the suggested outline or shorten the Introduction in a different way: 1. Motivate the need for an iterative in silico–in vivo approach. 2. Summarize the current state of the art and highlight what is missing. 3. Introduce the framework and emphasize its novelty. Comparisons with top-down models, as well as specific examples, might be better suited for the Discussion section (see the following comment). Along the same lines, consider revising the Abstract to provide a more concise overview of the work. Currently, it follows a similar structure to the Introduction. The decision to incorporate these stylistic changes is left at the authors' discretion.
We thank the reviewer for these suggestions to improve the style and clarity of our manuscript. We rewrote the abstract to provide a more concise overview of ISF, we shortened and rewrote parts of the Introduction, and we completely rewrote and extended the Discussion (see below).

Major 2. The Discussion section would benefit from a more focused comparison with existing modeling software. Currently, it reads as a general overview of the neuronal modeling field challenges rather than a targeted analysis of the proposed framework. The framework has both advantages and limitations compared to existing software. A comparison with relevant software and modeling approaches would provide valuable context for readers: Dura-Bernal et al., 2019 (NetPyNe), Dai et al., 2020 (BMTK), Reva et al., 2023, Roy and Narayanan 2023, Makarov et al., 2025 (DendroTweaks), Wybo 2025 (NEAT), Deistler et al., 2025 (Jaxley).
*We agree with the reviewer and rewrote the Discussion accordingly. We describe the general challenges that ISF seeks to address **(lines 376-421)**, what sets ISF apart from other bottom-up modelling approaches **(lines 422-440)**, the limitations of ISF **(lines 441-489)**, and how ISF may be combined with other frameworks **(lines 489-512)**.*

Major 3. The "neuron-network" term is potentially misleading, as it may imply that networks of multicompartmental biophysical neurons are explicitly modeled within the

framework. To avoid confusion, I suggest replacing the "neuron-network models" with "network-embedded neuron models" throughout the manuscript. This term is already used by the authors in the Methods and tutorials. Additionally, comparisons with multiscale network models, such as those described in Billeh et al. (2020) and Markram et al. (2015), may further contribute to this ambiguity and should be addressed carefully to maintain relevant scope.

We thank the reviewer for this suggestion and replaced neuron-network models with network-embedded neuron models throughout the manuscript, and in the Discussion, we now emphasize that we do not simulate dynamics in networks of multi-compartmental models ***(lines 481-494)****.*

Major 4. The Results and Methods sections don't clearly explain what the reduced model is. Is it still a biophysical model, or an abstract mapping from input to output? Does it involve morphological abstraction as a point neuron? The manuscript needs to clearly explain what results from the reduction.

We agree with the reviewer that the model reduction section was not sufficiently clear. The goal of the model reduction workflow in ISF is to derive analytically tractable models with a minimal set of parameters that can predict how the full models transform synaptic input into AP output. In the revised manuscript, we now introduce our concept of model reduction for specific in vivo conditions ***(lines 228-241)*** *and describe our approaches formally* ***(lines 723-743)****. In brief, ISF enables model reduction by sets of utility functions across three modules. (1) The <u>data_extractor</u> module enables users to specify which synaptic input features a reduced model should relate to which neuronal output features. (2) The <u>strategy</u> module enables users to define the mathematical forms of the inference functions that predict neuronal output from input. ISF provides example <u>Strategy</u> objects, such as GLMs, including a selection of basis functions (e.g. raised cosine). (3) The <u>solver</u> module enables users to define cost functions that capture how well the output produced by a reduced model matches the output of the multi-compartmental model (e.g. mean squared error or area under the receiver-operator curve). Furthermore, the <u>solver</u> module enables users to use built-in (or their own) optimization algorithms to identify reduced models that predict the full model output best. We describe how we had used the three modules for our example of L5PT responses in the barrel cortex* ***(lines 744-766)****, and provide it as an online tutorial* ***(<u>link</u>).***

Major 5. Consider making the code more configurable and less reliant on hardcoded data to ensure that the framework generalizes well to other models. Including model-specific code directly into the core packages raises questions about how well this pipeline extends to other models:

- **in_silico_framework/biophysics_fitting/L5tt_parameter_setup.py**
- **in_silico_framework/singlecell_input_mapper**
- **in_silico_framework/visualize/voltage_trace_visualizer.py**

Do not mix examples and the core package code:

- **in_silico_framework/biophysics_fitting/hay**
- **in_silico_framework/mechanisms**

Replace hardcoded functions with flexible, parameterized alternatives:

- **in_silico_framework/single_cell_parser/cell_modify_functions/soma_current_injection.py**
- **in_silico_framework/biophysics_fitting/setup_stim.py**

In tutorials, consider using preprocessed data to focus on the framework's functionality rather than data preparation:

- **scale_apical_morph_86**

Model-specific configurations in in_silico_framework/config are the correct approach, they are easy to replace and clearly communicate to the user what needs to be adjusted for a custom model. Consider separating configurations from source code elsewhere. This list of examples isn't exhaustive, but rather it is what became evident from an initial review of the codebase. If generalizability is already ensured, remove the hardcoded parameters

to avoid giving a false impression.
*We thank the reviewer for the thorough review of the source code and agree that making it more configurable and less reliant on hardcoded data is important. Therefore, we substantially revised the code and online tutorials to separate any L5PT and barrel cortex model-specific code from the core packages of ISF. For example, the network embedding module (**link**) now provides our strategy for the barrel cortex as a separate package, leaving the option to include other network embedding procedures – i.e., we now state in the revised manuscript **(lines 145-151)** and online resource (e.g. **link**) that ISF can be used with user-defined network embedding strategies. More specifically, we now specify the anatomical data that was necessary to generate the barrel cortex model **(link)**, we added a tutorial that explains contents and formats of these anatomical data **(link)**, linked this tutorial to the main tutorial for network embedding (**link**), we extended the online data format overview with a toggle that specifies the sources and kinds of these anatomical data (**link**), and we made our embedding procedure independent of hardcoded labels (**link**). **In sum**, the network embedding strategy is now left to the user, while we encourage and explain how to generate and use a dense neuropil model. Moreover, like our generalization of network embedding, we now also separated the other core functions of ISF from our specific examples and hardcoded parameters. For example, we moved L5tt_parameter_setup to the hay package, which is labelled now as an example implementation (**link**) for how to generate neuron models with the API of the biophysics_fitting module, whose other packages are general (**link**). Moreover, we moved the NEURON mechanisms to the user/config directory (**link**), replaced scale_apical_morph_86 with a general scale_apical function (**link**), removed old data writers that were specific to our projects, but no longer in use (**link**), highlight the requirements to use setup_apical_epsp_injection in the docstring (**link**), and ensured that all other major functions* are *parametrized and hence general – i.e., users pass in (e.g. in single_cell_parser/cell_modify_functions/soma_current_injection.py) the relevant parameters as arguments.*

Minor 1. The suggested workflow largely relies on BluePyOpt, making it important to clarify the framework's added value by comparing its data management capabilities to those of BluePyOpt.
*We thank the reviewer for pointing out this issue. It is indeed important to clarify that ISF does not depend BluePyOpt or any particular optimizer (e.g. in the past we had also used a HOC implementation of NSGA-II). In essence, ISF relies on the availability of a valid "seed" model that captures the desired empirically observed intrinsic physiology. For our example of L5PTs, BluePyOpt was most successful in identifying seed models that captured the complex dendritic and perisomatic physiology of this neuronal cell type. However, depending on the complexity of the neuron models, any other method that provides a seed model would compatible with ISF, including manual tuning or random sampling of parameters, or gradient-based optimization (e.g. via Jaxley). We now clarify this important point in the revised manuscript **(lines 597-604)**.*

Minor 2. The manuscript doesn't clarify which parameters can be modified and optimized e.g., whether distributions and kinetic parameters of ion channels can be changed, or only uniform channel conductances. The tutorials contain this information, but it might be beneficial to add it in the manuscript.
*We thank the reviewer for this suggestion. In the revised manuscript **(lines 616-633)** we clarify now that any parameter listed in the ISF neuron parameter specification can be modified, and hence be part of both optimization and exploration. More specifically, the neuron parameter specification of ISF can include parameters for spatial profiles of ion channel densities, as well as all NEURON parameters, including kinetic parameters, defined in the PARAMETER block of a loaded NMODL file. For channels, these parameters may include conductance densities for a given compartment. For calcium buffers, these may include the diffusion kinetics, such as the time constant, shell depth, and ratio of unbuffered calcium. In addition to any parameter of a NEURON mechanisms and spatial channel density parameters, users can specify additional parameters for custom functions under single_cell_parser/cell_modify_functions. We extended*

the parameter files documentation and how to select which parameters can be modified ***(link)***.

Minor 3. How would a channel distribution in oblique dendrites of a CA1 neuron be set, for example, given that a morphology file specifies them as part of the apical dendrite? *To set parameters in apical oblique dendrites that differ from those in other parts of the apical dendrite, the obliques need a different label (e.g. "ObliqueDendrite") in the morphology file. The channel distributions can then be defined by adding a key with the same label in the neuron parameters – i.e., keys in the neuron parameter file just need to match those in the morphology file. To further support the general use of ISF with custom labels in morphology files (now not only for HOC, but also for SWC files, see below), we extended user/config with a morphology label map* ***(link)****, where users can specify which morphology labels translate into which keys – e.g. ISF sets oblique dendrite-specific channel distributions (and parameters in general) to all compartments with the morphology label that is mapped to the key "ObliqueDendrite" in the neuron parameter file.*

Minor 4. The purpose of encapsulating Python packages under the Interface class (such as I.sns, I.shutil, I.os) is unclear. Consider making it more straightforward when relying on external packages, or provide utility functions to simplify setting up plots, paths, etc.
We thank the reviewer for this suggestion. However, encapsulating Python packages under the Interface class is deliberate choice that is based on our experience from using ISF. The purpose of making these external dependencies available through Interface is essentially to ensure API stability. Packages can (and often do) change API faster than the average life cycle of a large-scale project in ISF. The user may however still want to use new functionality, without losing API stability, and without having to rewrite analysis notebooks. By encapsulating important external dependencies in Interface this way, we allow the possibility of patching such upstream API changes for the duration of a project, which we did in the past for pandas and numpy.

Minor 5. Please clarify the rationale for using .hoc for morphology files instead of .swc. Additionally, what advantages does the custom .hoc reader provide compared to NEURON's default implementation?
We thank the reviewer for raising this question. HOC files with additional custom information (e.g. location within the barrel cortex) are the output of methods that we had developed to reconstruct in vivo-labelled neuron morphologies. Our custom reader also processes standard HOC files, and there are several tools available which transform neuron reconstructions into different file formats – from and to HOC. However, we agree that a SWC reader for ISF would facilitate general use. Thus, we now support reading SWC files in ISF and extended our online documentation ***(link)*** *and code accordingly* ***(link)****. We now also support converting from SWC to HOC (****link****) and back (****link****).*

Minor 6. The documentation would benefit from a simpler introductory tutorial. The configurations and setup might be better suited for a separate tutorial. An ideal introductory tutorial would clearly demonstrate the high-level workflow and core concepts of the framework. Splitting the code into smaller chunks with additional explanations between sections could improve clarity.
We agree with the reviewer and thank you for this suggestion. We removed the configuration step from the introductory tutorial. Configuration is now a subsection on the installation page ***(link)****. Moreover, we simplified the introductory tutorial by adding more subheadings, and by providing clearer explanations (****link****).*
