# Supplementary material for: An in silico framework for dissecting the mechanistic origins of in vivo recorded neuronal activity
Source: PLoS Comput Biol. 2026 Aug 21;22(8):e1014617. doi: 10.1371/journal.pcbi.1014617 (PMC13529221; doi:10.1371/journal.pcbi.1014617)
Supplement: Peer review history

# Peer review: Decision Letter 0

**DOI:** 10.1371/journal.pcbi.1014617.r001

**Authors:** Hugues Berry, Sacha Jennifer van Albada

10 Mar 2026

An in silico framework for modeling and analyzing in vivo neuron-network mechanisms

PLOS Computational Biology

Dear Dr. Oberlaender,

Thank you for submitting your manuscript to PLOS Computational Biology. After careful consideration, we feel that it has merit but does not fully meet PLOS Computational Biology's publication criteria as it currently stands. Therefore, we invite you to submit a revised version of the manuscript that addresses the points raised during the review process.

Please submit your revised manuscript by May 10 2026 11:59PM. If you will need more time than this to complete your revisions, please reply to this message or contact the journal office at ploscompbiol@plos.org. When you're ready to submit your revision, log on to https://www.editorialmanager.com/pcompbiol/ and select the 'Submissions Needing Revision' folder to locate your manuscript file.

Please include the following items when submitting your revised manuscript:

* A letter that responds to each point raised by the editor and reviewer(s). You should upload this letter as a separate file labeled 'Response to Reviewers'. This file does not need to include responses to formatting updates and technical items listed in the 'Journal Requirements' section below.

* A marked-up copy of your manuscript that highlights changes made to the original version. You should upload this as a separate file labeled 'Revised Manuscript with Track Changes'.

* An unmarked version of your revised paper without tracked changes. You should upload this as a separate file labeled 'Manuscript'.

If you would like to make changes to your financial disclosure, competing interests statement, or data availability statement, please make these updates within the submission form at the time of resubmission. Guidelines for resubmitting your figure files are available below the reviewer comments at the end of this letter

We look forward to receiving your revised manuscript.

Kind regards,

Sacha Jennifer van Albada

Academic Editor

PLOS Computational Biology

Hugues Berry

Section Editor

PLOS Computational Biology

**Journal Requirements:**

1) Please ensure that the CRediT author contributions listed for every co-author are completed accurately and in full.

At this stage, the following Authors/Authors require contributions: Bjorge Meulemeester, Arco Bast, Maria Royo, Rieke Fruengel, and Marcel Oberlaender. Please ensure that the full contributions of each author are acknowledged in the "Add/Edit/Remove Authors" section of our submission form.

The list of CRediT author contributions may be found here: https://journals.plos.org/ploscompbiol/s/authorship#loc-author-contributions

2) We ask that a manuscript source file is provided at Revision. Please upload your manuscript file as a .doc, .docx, .rtf or .tex. If you are providing a .tex file, please upload it under the item type u2018LaTeX Source Fileu2019 and leave your .pdf version as the item type u2018Manuscriptu2019.

3) Please confirm whether your study includes live participants, If so, please insert an Ethics Statement at the beginning of your Methods section, under a subheading 'Ethics Statement'. It must include:

i) The full name(s) of the Institutional Review Board(s) or Ethics Committee(s)

ii) The approval number(s), or a statement that approval was granted by the named board(s).

4) Please upload all main figures as separate Figure files in .tif or .eps format. For more information about how to convert and format your figure files please see our guidelines:

https://journals.plos.org/ploscompbiol/s/figures

5) We notice that your supplementary Figures are included in the manuscript file. Please remove them and upload them with the file type 'Supporting Information'. Please ensure that each Supporting Information file has a legend listed in the manuscript after the references list.

6) Some material included in your submission may be copyrighted. According to PLOSu2019s copyright policy, authors who use figures or other material (e.g., graphics, clipart, maps) from another author or copyright holder must demonstrate or obtain permission to publish this material under the Creative Commons Attribution 4.0 International (CC BY 4.0) License used by PLOS journals. Please closely review the details of PLOSu2019s copyright requirements here: PLOS Licenses and Copyright. If you need to request permissions from a copyright holder, you may use PLOS's Copyright Content Permission form.

Please respond directly to this email and provide any known details concerning your material's license terms and permissions required for reuse, even if you have not yet obtained copyright permissions or are unsure of your material's copyright compatibility. Once you have responded and addressed all other outstanding technical requirements, you may resubmit your manuscript within Editorial Manager.

Potential Copyright Issues:

i) Figures 5C (Panel adapted from Udvary et al. (2022) [33]), 5I, and 5J ( Panel adapted from Egger et al. (2020) [21). Please note that the published papers are licensed under CC BY-NC-ND which is not compatible with our CC BY 4.0 license. Please provide written permission from the copyright holder to publish this under our CC-BY 4.0 license, or remove the figure / replace the image. Please note we do not recommend using standard request forms available on Publishers' websites, as they grant single use rather than republication under an open access license.

7) Please amend your detailed Financial Disclosure statement. This is published with the article. It must therefore be completed in full sentences and contain the exact wording you wish to be published.

1) State what role the funders took in the study. If the funders had no role in your study, please state: "The funders had no role in study design, data collection and analysis, decision to publish, or preparation of the manuscript."

8)  Please ensure that the funders and grant numbers match between the Financial Disclosure field and the Funding Information tab in your submission form. Note that the funders must be provided in the same order in both places as well.

Note: If the reviewer comments include a recommendation to cite specific previously published works, please review and evaluate these publications to determine whether they are relevant and should be cited. There is no requirement to cite these works unless the editor has indicated otherwise.

**Reviewers' comments:**

Reviewer's Responses to Questions

Reviewer #1: This manuscript presents the In Silico Framework (ISF), an open-source environment for generating and analyzing ensembles of biologically detailed neuron-network models. The central conceptual contribution, shifting the focus from single “tuned” models to diverse ensembles that capture biological degeneracy, is a timely and significant step for computational neuroscience. The integration of multi-scale data to achieve “model consensus” is a powerful approach for making testable in vivo predictions. The framework is technically impressive and well-demonstrated through its application to the rat barrel cortex. I support publication, as ISF aligns perfectly with the scope of PLOS Computational Biology. However, the manuscript would benefit from several specific clarifications to help potential users understand the practical deployment and limitations of the tool.

Minor Revisions / Clarifications:

Data Requirements & Accessibility:

While the manuscript notes that statistical methods can be used to generate neuropil models as an alternative to dense EM reconstructions, many researchers may lack such resources or pre-established statistical models. Could the authors more explicitly define the “minimum viable data” required to use ISF? Even a short schematic outlining required versus optional inputs would greatly increase accessibility for potential adopters.

Computational Cost:

To help the community assess the feasibility of adopting ISF, it would be helpful to provide approximate estimates of computational requirements (e.g., CPU-hours, peak memory, and storage) for a representative workflow. Even order-of-magnitude estimates would be informative for groups without access to large-scale computational infrastructure.

Troubleshooting “Model Failure”:

The authors state that if no model reproduces an in vivo observation, the generation process requires further refinement. This is a critical juncture for any user. Could the authors expand on the recommended diagnostic workflow in such cases? For instance, how might a researcher systematically distinguish between a missing biological mechanism versus an insufficiently explored parameter space or overly restrictive empirical constraints? Providing guidance on this would strengthen the methodological transparency of the framework.

Mathematical Form of Reduced Models:

The model reduction section remains somewhat abstract. While GLMs are mentioned in the example application, it is unclear what “minimal set of parameters” means in the general case. Does ISF provide a library of reduction methods (e.g., GLMs, kernel-based approaches, specific filter architectures), or is the user expected to define the mathematical form entirely? A clearer description of the interface for implementing reduced models, and what is provided out of the box, would significantly improve clarity in this important conceptual component of the framework.

Overall, I believe this manuscript represents a valuable and timely contribution. With clarification of the above points, it will serve as an important resource for researchers interested in ensemble-based, mechanistic modeling of in vivo neural activity.

Reviewer #2: Summary:

The manuscript by Meulemeester et al. introduces the In-Silico Framework (ISF) for iterative investigation of single neuron function, bridging in silico predictions with in vivo observations. The proposed framework is a valuable contribution to the field, as it offers a way to integrate the expanding body of experimental data to advance mechanistic understanding of neural activity in vivo. The authors provide compelling examples of the framework’s application based on their prior research. However, the generalizability of the framework is not immediately clear from the manuscript or the provided code. That said, the manuscript is well-written and visually engaging, with clear illustrations. The accompanying code is publicly available, well-documented, and includes helpful tutorials.

Positive aspects:

1. The proposed workflow effectively bridges in silico simulations and in vivo observations, demonstrating a well-structured and systematic approach. It incorporates rigorous sensitivity analysis and parameter exploration, enabling targeted manipulations and identifying key mechanisms of neuronal computations.

2. The provided examples are highly compelling and provide solid evidence of the predictive power of the framework.

2. The visuals produced by the package are of high quality.

3. Performance considerations are addressed by the use of scalable tools such as Dask, ensuring efficiency and applicability to large datasets.

Major comments:

1. I recommend balancing the content of the Introduction and Discussion sections. The authors might follow the suggested outline or shorten the Introduction in a different way:

¶1: Motivate the need for an iterative in silico–in vivo approach.

¶2: Summarize the current state of the art and highlight what is missing.

¶3: Introduce the framework and emphasize its novelty.

Comparisons with top-down models, as well as specific examples, might be better suited for the Discussion section (see the following comment). Along the same lines, consider revising the Abstract to provide a more concise overview of the work. Currently, it follows a simular structure to the Introduction. The decision to incorporate these stylistic changes is left at the authors' discretion.

2. The Discussion section would benefit from a more focused comparison with existing modeling software. Currently, it reads as a general overview of the neuronal modeling field challenges rather than a targeted analysis of the proposed framework. The framework has both advantages and limitations compared to existing software. A comparison with relevant software and modeling approaches would provide valuable context for readers:

Dura-Bernal et al., 2019 https://doi.org/10.7554/eLife.44494 (NetPyNe),

Dai et al., 2020 https://doi.org/10.1371/journal.pcbi.1008386 (BMTK),

Reva et al., 2023 https://doi.org/10.1016/j.patter.2023.100855,

Roy and Narayanan 2023 https://doi.org/10.1113/JP283539,

Makarov et al., 2025 https://doi.org/10.7554/eLife.103324.3 (DendroTweaks),

Wybo 2025 https://doi.org/10.1101/2025.06.26.661734 (NEAT),

Deistler et al., 2025 https://doi.org/10.1038/s41592-025-02895-w (Jaxley).

3. The “neuron-network” term is potentially misleading, as it may imply that networks of multicompartmental biophysical neurons are explicitly modeled within the framework. To avoid confusion, I suggest replacing the “neuron-network models” with “network-embedded neuron models” throughout the manuscript. This term is already used by the authors in the Methods and tutorials. Additionally, comparisons with multiscale network models, such as those described in Billeh et al. (2020) and Markram et al. (2015), may further contribute to this ambiguity and should be addressed carefully to maintain relevant scope.

4. The Results and Methods sections don't clearly explain what the reduced model is. Is it still a biophysical model, or an abstract mapping from input to output? Does it involve morphological abstraction as a point neuron? The manuscript needs to clearly explain what results from the reduction.

5. Consider making the code more configurable and less reliant on hardcoded data to ensure that the framework generalizes well to other models. Including model-specific code directly into the core packages raises questions about how well this pipeline extends to other models:

- in_silico_framework/biophysics_fitting/L5tt_parameter_setup.py

- in_silico_framework/singlecell_input_mapper

- in_silico_framework/visualize/voltage_trace_visualizer.py

Do not mix examples and the core package code:

- in_silico_framework/biophysics_fitting/hay

- in_silico_framework/mechanisms

Replace hardcoded functions with flexible, parameterized alternatives:

- in_silico_framework/single_cell_parser/cell_modify_functions/soma_current_injection.py

- in_silico_framework/biophysics_fitting/setup_stim.py

In tutorials, consider using preprocessed data to focus on the framework's functionality rather than data preparation:

- scale_apical_morph_86

Model-specific configurations in in_silico_framework/config are the correct approach, they are easy to replace and clearly communicate to the user what needs to be adjusted for a custom model. Consider separating configurations from source code elsewhere.

This list of examples isn't exhaustive, but rather it is what became evident from an initial review of the codebase. If generalizability is already ensured, remove the hardcoded parameters to avoid giving a false impression.

Minor comments:

1. The suggested workflow largely relies on BluePyOpt, making it important to clarify the framework’s added value by comparing its data management capabilities to those of BluePyOpt.

2. The manuscript doesn't clarify which parameters can be modified and optimized e.g., whether distributions and kinetic parameters of ion channels can be changed, or only uniform channel conductances. The tutorials contain this information, but it might be beneficial to add it in the manuscript.

3. How would a channel distribution in oblique dendrites of a CA1 neuron be set, for example, given that a morphology file specifies them as part of the apical dendrite?

4. The purpose of encapsulating Python packages under the Interface class (such as I.sns, I.shutil, I.os) is unclear. Consider making it more straightforward when relying on external packages, or provide utility functions to simplify setting up plots, paths, etc.

5. Please clarify the rationale for using .hoc for morphology files instead of .swc. Additionally, what advantages does the custom .hoc reader provide compared to NEURON's default implementation?

6. The documentation would benefit from a simpler introductory tutorial. The configurations and setup might be better suited for a separate tutorial. An ideal introductory tutorial would clearly demonstrate the high-level workflow and core concepts of the framework. Splitting the code into smaller chunks with additional explanations between sections could improve clarity.

**********

**Have the authors made all data and (if applicable) computational code underlying the findings in their manuscript fully available?**

The PLOS Data policy  requires authors to make all data and code underlying the findings described in their manuscript fully available without restriction, with rare exception (please refer to the Data Availability Statement in the manuscript PDF file). The data and code should be provided as part of the manuscript or its supporting information, or deposited to a public repository. For example, in addition to summary statistics, the data points behind means, medians and variance measures should be available. If there are restrictions on publicly sharing data or code —e.g. participant privacy or use of data from a third party—those must be specified.

Reviewer #1: None

Reviewer #2: Yes

**********

PLOS authors have the option to publish the peer review history of their article (what does this mean? ). If published, this will include your full peer review and any attached files.

If you choose “no”, your identity will remain anonymous but your review may still be made public.

**Do you want your identity to be public for this peer review?**   For information about this choice, including consent withdrawal, please see our Privacy Policy .

Reviewer #1: No

Reviewer #2: No

[NOTE: If reviewer comments were submitted as an attachment file, they will be attached to this email and accessible via the submission site. Please log into your account, locate the manuscript record, and check for the action link "View Attachments". If this link does not appear, there are no attachment files.]

**Figure resubmission:**

After uploading your figures to PLOS’s NAAS tool - https://ngplosjournals.pagemajik.ai/artanalysis, NAAS will process the files provided and display the results in the "Uploaded Files" section of the page as the processing is complete. If the uploaded figures meet our requirements (or NAAS is able to fix the files to meet our requirements), the figure will be marked as "fixed" above. If NAAS is unable to fix the files, a red "failed" label will appear above. When NAAS has confirmed that the figure files meet our requirements, please download the file via the download option, and include these NAAS processed figure files when submitting your revised manuscript.

**Reproducibility:**

To enhance the reproducibility of your results, we recommend that authors of applicable studies deposit laboratory protocols in protocols.io, where a protocol can be assigned its own identifier (DOI) such that it can be cited independently in the future. Additionally, PLOS ONE offers an option to publish peer-reviewed clinical study protocols. Read more information on sharing protocols at https://plos.org/protocols?utm_medium=editorial-email&utm_source=authorletters&utm_campaign=protocols

# Author comment: Author response to Decision Letter 1

**DOI:** 10.1371/journal.pcbi.1014617.r002

11 Jun 2026

Attachment Submitted filename: MeulemeesterEtAl_RebuttalLetter_R1.pdf

# Peer review: Decision Letter 1

**DOI:** 10.1371/journal.pcbi.1014617.r003

**Authors:** Hugues Berry, Sacha Jennifer van Albada

22 Jul 2026

Dear Prof. Dr. Oberlaender,

We are pleased to inform you that your manuscript 'An in silico framework for dissecting the mechanistic origins of in vivo recorded neuronal activity' has been provisionally accepted for publication in PLOS Computational Biology.

Before your manuscript can be formally accepted you will need to complete some formatting changes, which you will receive in a follow up email. A member of our team will be in touch with a set of requests.

Please note that your manuscript will not be scheduled for publication until you have made the required changes, so a swift response is appreciated.

IMPORTANT: The editorial review process is now complete. PLOS will only permit corrections to spelling, formatting or significant scientific errors from this point onwards. Requests for major changes, or any which affect the scientific understanding of your work, will cause delays to the publication date of your manuscript.

Should you, your institution's press office or the journal office choose to press release your paper, you will automatically be opted out of early publication. We ask that you notify us now if you or your institution is planning to press release the article. All press must be co-ordinated with PLOS.

Thank you again for supporting Open Access publishing; we are looking forward to publishing your work in PLOS Computational Biology.

Best regards,

Sacha Jennifer van Albada

Academic Editor

PLOS Computational Biology

Hugues Berry

Section Editor

PLOS Computational Biology

***********************************************************

Reviewer's Responses to Questions

**Comments to the Authors:**

**Please note here if the review is uploaded as an attachment.**

Reviewer #2: The authors have improved the quality of the manuscript and the source code and have successfully addressed the comments from the previous round of revisions. In its current state, the In Silico Framework (ISF) has the potential to improve neuronal models and generate testable predictions for in vivo experiments, and it should be further refined through community feedback. I therefore support publication of the manuscript in PLOS Computational Biology as a valuable contribution to the field.

**********

**Have the authors made all data and (if applicable) computational code underlying the findings in their manuscript fully available?**

The PLOS Data policy  requires authors to make all data and code underlying the findings described in their manuscript fully available without restriction, with rare exception (please refer to the Data Availability Statement in the manuscript PDF file). The data and code should be provided as part of the manuscript or its supporting information, or deposited to a public repository. For example, in addition to summary statistics, the data points behind means, medians and variance measures should be available. If there are restrictions on publicly sharing data or code —e.g. participant privacy or use of data from a third party—those must be specified.

Reviewer #2: Yes

**********

PLOS authors have the option to publish the peer review history of their article (what does this mean? ). If published, this will include your full peer review and any attached files.

If you choose “no”, your identity will remain anonymous but your review may still be made public.

**Do you want your identity to be public for this peer review?**   For information about this choice, including consent withdrawal, please see our Privacy Policy .

Reviewer #2: No

# Editor report: Acceptance letter

**DOI:** 10.1371/journal.pcbi.1014617.r004

**Authors:** Hugues Berry, Sacha Jennifer van Albada

PCOMPBIOL-D-26-00095R1

An in silico framework for dissecting the mechanistic origins of in vivo recorded neuronal activity

Dear Dr Oberlaender,

I am pleased to inform you that your manuscript has been formally accepted for publication in PLOS Computational Biology. Your manuscript is now with our production department and you will be notified of the publication date in due course.

The corresponding author will soon be receiving a typeset proof for review, to ensure errors have not been introduced during production. Please review the PDF proof of your manuscript carefully, as this is the last chance to correct any errors. Please note that major changes, or those which affect the scientific understanding of the work, will likely cause delays to the publication date of your manuscript.

Soon after your final files are uploaded, unless you have opted out, the early version of your manuscript will be published online. The date of the early version will be your article's publication date. The final article will be published to the same URL, and all versions of the paper will be accessible to readers.

For Research, Software, and Methods articles, you will receive an invoice from PLOS for your publication fee after your manuscript has reached the completed accept phase. If you receive an email requesting payment before acceptance or for any other service, this may be a phishing scheme. Learn how to identify phishing emails and protect your accounts at https://explore.plos.org/phishing.

Thank you again for supporting PLOS Computational Biology and open-access publishing. We are looking forward to publishing your work!

With kind regards,

Sharmila Kamatchi

PLOS Computational Biology | Carlyle House, Carlyle Road, Cambridge CB4 3DN | United Kingdom ploscompbiol@plos.org | Phone +44 (0) 1223-442824 | ploscompbiol.org | @PLOSCompBiol
